# RHBDL2 promotes the proliferation, migration, and invasion of pancreatic cancer by stabilizing the N1ICD via the OTUD7B and activating the Notch signaling pathway

**DOI:** 10.1038/s41419-022-05379-3

**Published:** 2022-11-09

**Authors:** Shiyu Chen, Kun Cai, Dijie Zheng, Yanqing Liu, Lin Li, Zhiwei He, Chengyi Sun, Chao Yu

**Affiliations:** 1 Department of Hepatobiliary Surgery, The Affiliated Hospital of Guizhou Medical University, Guiyang, Guizhou 550004 China; 2 Guizhou Provincial Institute of Hepatobiliary, Pancreatic and Splenic Diseases, Guiyang, Guizhou 550004 China; 3 Guizhou Provincial Clinical Medical Research Center of Hepatobiliary Surgery, Guiyang, Guizhou 550004 China; 4 Key Laboratory of Liver, Gallbladder, Pancreas and Spleen of Guizhou Medical University, Guiyang, Guizhou 550004 China; 5 School of Basic Medical Sciences, Guizhou Medical University, Guiyang, Guizhou 550004 China; 6 Department of Translational Medicine, College of Clinical Medicine, Guizhou Medical University, Guiyang, Guizhou 550004 China; 7 Guizhou Medical University, Guiyang, Guizhou 550004 China; 8 Department of Surgery, College of Clinical Medicine, Guizhou Medical University, Guiyang, Guizhou 550004 China

**Keywords:** Cancer, Molecular biology

## Abstract

Pancreatic cancer (PC) is one of the most malignant types of cancer, and is characterized by early metastasis, limited response to chemotherapeutics, and poor prognosis. Therefore, there is an urgent need to explore new therapeutic strategies for PC treatment. Human rhomboid-like 2 (RHBDL2) is differentially expressed in cervical and breast cancer. However, the correlation between RHBDL2 and PC remains unclear. We found that RHBDL2 is highly expressed in human PC cells and tissues and is significantly associated with distant metastasis and poor survival of patients with PC. Gain- and loss-of-function assays indicated that RHBDL2 could accelerate PC cell proliferation and mobility in vitro and in vivo. The RNA-Seq results suggest that RHBDL2 may be involved in the activation of Notch signaling pathway. IMR-1 could restore the proliferation and metastatic capacity of PC cells mediated by RHBDL2. RHBDL2 interacted with and cleaved Notch1, resulting in the release of N1ICD. RHBDL2 decreased the ubiquitination level of N1ICD and collaborated with Ovarian tumor domain-containing 7B (OTUD7B) to stabilize N1ICD via the ubiquitin-proteasome pathway. RHBDL2 facilitated PC cell proliferation and mobility by stabilizing the N1ICD via the OTUD7B and activating the Notch signaling pathway. Thus, targeting this novel pathway may be a potential therapeutic strategy for PC.

## Introduction

Pancreatic cancer (PC) is one of the most malignant types of cancer and is characterized by local invasion, early metastasis, and limited response to chemotherapeutics [1, 2]. These dismal characteristics lead to poor prognosis in PC patients, with a low 5-year overall survival rate (less than 5%) [3]. Hence, understanding the biological processes and underlying molecular mechanisms of PC may aid in the development of effective therapeutic methods.

Human rhomboid-like 2 (RHBDL2) is a member of the rhomboid family of the integral membrane proteins and is associated with Drosophila rhomboid proteins [4, 5]. RHBDL2 functions as an intramembrane serine protease, which can cleave the membrane-anchored DER ligand Spitz [6], the collagen receptor tyrosine kinase DDR1 [7], Ca^2+^ release-activated Ca^2+^ (CRAC) channels pore-forming subunit Orai1 [8], and so forth. Koch et al. identified RHBDL2 as an alternative sheddase that can efficiently induce IL-11R secretion. In addition, they determined the RHBDL2 cleavage site, which is close to the plasma membrane, between Ala-370 and Ser-371 [9]. RHBDL2 mRNA expression is markedly elevated in low-grade breast cancer tissues compared with that in normal breast samples [10]. Cheng et al. reported that RHBDL2 is overexpressed in the breast cancer cell line MDA-MB-231 and cervical cancer cell line HeLa S3, which can cleave the EGF ligand and thereby result in the activation of EGFR signaling, finally leading to cell proliferation and reduction in cell adhesion [11]. However, to date, the impact of RHBDL2 on PC progression and its clinical and survival significance have not been clearly elucidated.

The Notch signaling pathway modulates various biological processes including carcinogenesis [12**–**14]. Once the Notch receptor binds to the ligand, it is cleaved, leading to the release of the Notch intracellular domain (NICD). The NICD is translocated to the nucleus, where it binds to the transcription complex CSL/RBPJκ. Thereafter, the transcription of downstream target genes is activated, which further accelerates cancer progression, including PC [15**–**17]. Notch1 is a Notch receptor and is dysregulated in a variety of cancer types [18**–**20]; however, the underlying mechanism between Notch1 and PC needs to be studied further.

Ubiquitination is a vital process involved in the progression of various cancer types, including PC [21**–**23]. Deubiquitinase (DUB) belongs to a large group of enzymes that can remove ubiquitin chains from specific proteins and thereby stabilize them [24]. To date, DUBs have a significant role in the regulation of DNA replication stress [25], inflammasome activation [26], and cancer progression [27**–**29]. Ovarian tumor domain-containing 7B (OTUD7B) is a DUB of the OTU protein superfamily and plays an important role in the development of lung cancer [30], breast cancer [31], and hepatocellular carcinoma [32]. However, whether OTUD7B can function as a DUB to modulate the Notch signaling pathway in PC remains unknown.

The present study found that RHBDL2 is highly expressed in PC cells and tissues, and can promote the proliferation, migration, and invasion capacity of PC cells in vitro and in vivo by activating the Notch signaling pathway. RHBDL2 interacts with Notch1 and mediates the cleavage and release of Notch1 intracellular domain (N1ICD). RHBDL2 inhibits the degradation of N1ICD through the ubiquitin-proteasome pathway. OTUD7B was identified as a DUB that interacts with both RHBDL2 and N1ICD and decreases the ubiquitination level of N1ICD. RHBDL2 collaborates with OTUD7B to stabilize N1ICD. The role of RHBDL2 in the proliferation, migration, and invasion of PC cells relies on its protease-cleavage activity and OTUD7B’s DUB function. The present study identified a new posttranslational modulation mechanism of N1ICD mediated by RHBDL2 and OTUD7B, which may contribute to the identification of efficient treatment strategies for PC.

## Methods

### Patients and specimens

Surgical specimens of PC and adjacent non-tumor tissues were acquired from 95 patients who underwent surgical resection for PC at the Department of Hepatobiliary Surgery, the Affiliated Hospital of Guizhou Medical University. This study was approved by the ethics committee of the Affiliated Hospital of Guizhou Medical University (2018 Lunshen No.018), and each patient provided written informed consent.

### Cell culture and chemicals

Antibodies were obtained from Abcam (Cambridge, MA, USA), Proteintech (Wuhan, Hubei, China), and Cell Signaling Technology (Danvers, MA, USA). Human pancreatic ductal epithelial (HPDE) cells and PC cell lines (CFPAC-1, MIA PaCa-2, SW1990, AsPC-1, PANC-1, and BxPC-3) were purchased from American Type Culture Collection (Manassas, VA, USA). These cell lines were authenticated via STR profiling and confirmed to be mycoplasma-free. The cell lines were grown in Dulbecco’s Modified Eagle Medium or Roswell Park Memorial Institute Medium (Gibco, Waltham, MA, USA) supplemented with 1% penicillin/streptomycin (Gibco) and 10% fetal bovine serum (Gibco) at 37 °C in a 5% CO_2_ humidified atmosphere. For all of the cellular experiments, three independent repeated assays were performed. Small molecular compounds, including IMR-1 (Selleck), MG-132 (Selleck), 3-methyladenine (3-MA; Selleck), cycloheximide (CHX; Sigma), chloroquine (Sigma), and NH_4_Cl (Aladdin), were purchased from the indicated suppliers.

### RNA extraction and quantitative real-time polymerase chain reaction

TRIzol reagent (Invitrogen, Carlsbad, CA, USA) was used to extract RNA from cells and tissues. cDNA was obtained using PrimeScript RT reagent (TaKaRa, Dalian, China) to conduct reverse transcription. A quantitative real-time polymerase chain reaction (qRT-PCR) assay was performed to investigate the gene expression levels.

### Western blot assay

Radioimmunoprecipitation assay buffer (Thermo Fisher Scientific; Waltham, MA, USA) mixed with protease inhibitors (Boster Biological Technology; Wuhan, Hubei, China) was used to extract proteins from cells and tissues. A bicinchoninic acid (BCA) assay kit (Biosharp, Hefei, Anhui, China) was used to quantify the protein. Sodium dodecyl sulfate polyacrylamide gel electrophoresis (SDS-PAGE) was performed to separate 40 mg of total protein. Specific antibodies were used to probe blots. Three independent repeated assays were performed.

### siRNAs and plasmid transfection and lentivirus infection

Small interfering RNAs (siRNAs) targeting RHBDL2, ATXN3, OTUD7B, and USP10 were acquired from RiboBio (Guangzhou, China). Plasmids for Myc-tagged N1ICD and its truncations, Flag-tagged wild-type RHBDL2, Flag-tagged RHBDL2-SA mutant, HA-tagged ubiquitin, HA-tagged OTUD7B, Flag-tagged OTUD7B, and Flag-tagged OTUD7B-C194S mutant were purchased from WZ Biosciences, Inc. (Shandong, China). The RHBDL2 and short hairpin RNA lentiviruses were purchased from GeneChem (Shanghai, China). siRNA and plasmid transfection were conducted using Lipofectamine 3000 (Invitrogen, Waltham, MA, USA) according to the manufacturer’s instructions. After infecting the PC cells with lentivirus for 48 h, stable cell lines were selected for 2 weeks and cultured with puromycin (1 μM, Invitrogen).

### Immunohistochemistry

Tissue sections were deparaffinized with xylene and rehydrated using an ethanol gradient. The samples were incubated with sodium citrate for antigen retrieval, and goat serum was used to block non-specific binding sites. Thereafter, primary antibodies, including Ki-67 (1:2000, Proteintech, 27309-1-AP), RHBDL2 (1:200, Proteintech, 12467-1-AP), and PCNA (1:200, Proteintech, 10205-2-AP), were incubated at 4 °C overnight, and subsequently incubated with the corresponding secondary antibodies for 2 h at room temperature. The positively stained cells and signal intensity in three randomly selected areas were examined and scored blindly by two independent observers.

### Cell Counting Kit-8 assay

PC cells (3000/well) were seeded in a 96-well plate and cultured for 0, 24, 48 and 72 h, respectively. Thereafter, 110 μl of mixed solution (10 μl of Cell Counting Kit-8 [CCK-8] reagent plus 100 μl of complete medium) was added to the wells in accordance with the manufacturer’s instructions (Dojindo Molecular Technologies, Inc., Japan). After incubating for 2 h, a microplate reader (Tecan, Austria) was used to determine the absorbance at a wavelength of 450 nm. Five replicate wells per group and three independent repeated assays were performed.

### Colony formation assay

The PC cells were placed in 6-well plates (800 cells/well) and incubated for two weeks. The cells were fixed with 4% paraformaldehyde (Biosharp, Hefei, Anhui, China) for 20 min. After washing with phosphate-buffered saline (PBS), 0.25% crystal violet solution (Biosharp, Hefei, Anhui, China) was used to stain cells for another 20 min. Finally, the culture plates were photographed.

### EdU assay

The PC cells were seeded in 24-well culture plates covered with slides and cultured for 24 h. Subsequently, 10 μM of EdU solution was added to the cells and incubated for 24 h. The cells were fixed with 4% paraformaldehyde for 20 min and washed with PBS. After permeabilization with 0.5% Triton X-100 for 10 min, the cells were stained with Apollo 567 and Hoechst 33342. An Olympus FSX100 microscope (Olympus, Tokyo, Japan) was used to capture the images.

### Wound healing assay

The PC cells were seeded in 6-well plates. When the cells reached confluence, a 200-μl pipette tip was used to scratch the wound. The cells were then incubated with serum-free medium for 48 h. The images of each wound were captured using an inverted microscope (Olympus, Tokyo, Japan) at 0 h and 48 h after scraping.

### Transwell assays

The cells that underwent starvation treatment were plated in the upper chamber, while 700 μl of complete medium was added to the bottom chamber. After culturing for 24 h, the cells in the upper chamber were removed. The cells were fixed or stained with 4% paraformaldehyde and 0.25% crystal violet solution. Matrigel was used to coat the upper membrane prior to cell seeding to analyze cell invasion. Migrated and invasive cells were imaged using a microscope.

### Animal assays

A total of 25 female 6-week-old BALB/c nude mice were randomly divided into five groups. The PC cells (2 × 10^6^/mouse) were injected subcutaneously into the right armpit of the mice. When the tumors became macroscopically visible, calipers were used to monitor them weekly. The equation (length × width^2^)/2 was used to calculate the tumor volume. After 42 days, the tumors were excised, fixed with 4% paraformaldehyde, and embedded in paraffin for IHC. For metastasis analysis, the PANC-1 cells (1 × 10^6^/mouse) were injected into the tail vein of the mice. After 56 days, the mice were euthanized to excise the lungs, and the number of pulmonary metastatic nodules was counted and further validated through HE staining. All animal studies were approved by the Animal Care Welfare Committee of Guizhou Medical University (No. 1801105).

### Dual Luciferase Reporter Assay

The cells were cultured in 6-well plates overnight, and the plasmids were subsequently transfected into the cells using Lipofectamine 3000 reagent (Invitrogen, Waltham, MA, USA) in accordance with the manufacturer’s instructions. Subsequently, the cells were lyzed, and the Dual Luciferase Reporter Assay Kit (Promega, Madison, WI, USA) was used to determine the firefly and renilla luciferase signals.

### Immunoprecipitation, silver staining, and mass spectrometry

PC cells were lyzed, and proteins were harvested and incubated with the RHBDL2 primary antibody overnight at 4 °C on a rotating wheel. Subsequently, Sepharose-conjugated protein A + G beads (Beyotime, Shanghai, China) were added to the mixtures and rotated for 4 h at 4 °C. Later, the beads were collected after full centrifugation and washing, and were boiled to isolate the protein. SDS-PAGE assay was performed for further investigation, and the proteins were subjected to Western blotting, silver staining, or mass spectrometry (MS) analysis. Silver staining assay was conducted using the Silver Stain Detection Kit in accordance with the manufacturer’s instructions. MS was performed at Genecreate Inc. (Wuhan, Hubei, China).

### Immunofluorescence assay

The PC cells were seeded in 24-well plates covered with slides and cultured for 24 h. Next, 4% paraformaldehyde and 0.5% Triton X-100 solution were used to fix and permeabilize the cells, respectively. Non-specific binding sites were blocked with 5% bovine serum albumin. The cells were incubated with primary antibodies against RHBDL2 (1:200, Proteintech, 12467-1-AP) and Notch1 (1:200, Proteintech, 10062-2-AP) at 4 °C for 16 h. After washing with PBS, the cells were incubated with the corresponding fluorescent secondary antibodies in the dark for 2 h at room temperature. After washing with PBS, 4′, 6-diamidino-2-phenylindole was used to stain the cell nuclei. A fluorescence microscope was used to detect the target protein expression.

### Statistical analysis

The results were analyzed using SPSS (version 23.0; IBM Corp., Armonk, NY, USA), and quantitative data were expressed as mean ± standard deviation. Student’s *t*-test (two-tailed) was used to examine the differences between two samples, while multiple groups were evaluated using a one-way analysis of variance. The overall survival was determined using the Kaplan-Meier method. A *P* value of <0.05 was considered significant.

## Results

### Overexpression of RHBDL2 and its positive correlation with poor prognosis in patients with PC

**Fig. 1:**
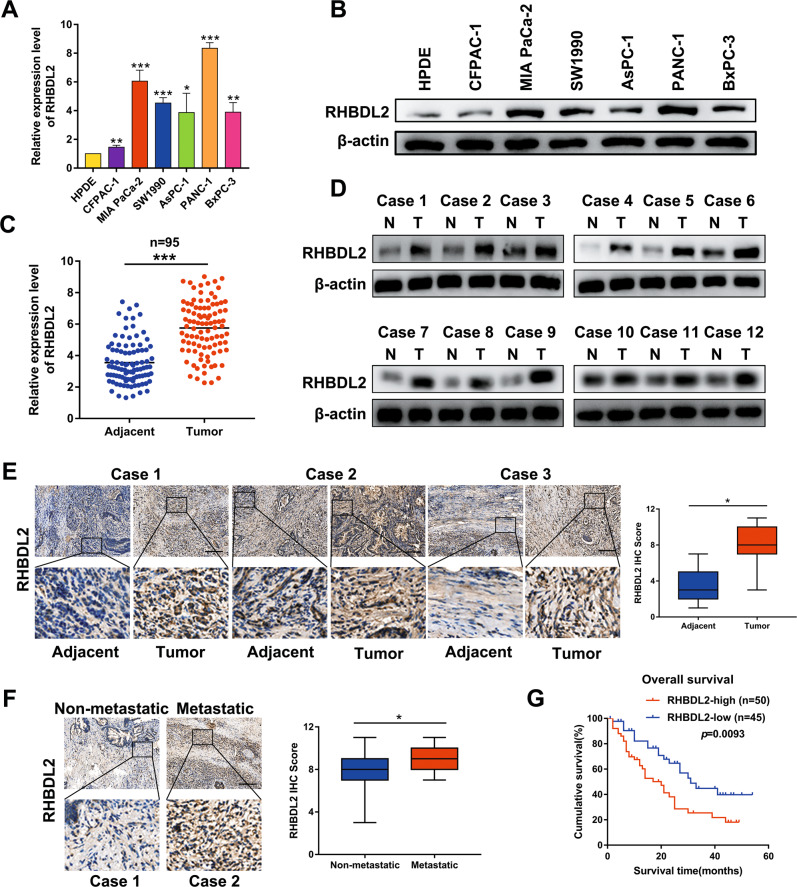
RHBDL2 is upregulated in PC and positively related to worse prognosis of PC patients. **A**, **B** Expression of RHBDL2 in the HPDE cells and six PC cell lines (CFPAC-1, MIA PaCa-2, SW1990, AsPC-1, PANC-1, and BxPC-3) was determined by qRT-PCR and Western blot assays, respectively. **C**, **D** qRT-PCR and Western blot analysis of RHBDL2 expression in PC tissues and adjacent non-tumor tissues. IHC assay confirmed the levels of RHBDL2 expression in the PC tissues compared with that in the adjacent non-tumor tissues (**E**), and in metastatic PC tissues compared with that in non-metastatic PC tissues (**F**), respectively (*n* = 95). Scale bar: 100 μm. **G** Kaplan-Meier analysis of PC patients with high/low RHBDL2 expression. *P < 0.05, **P < 0.01, ***P < 0.001.

By analyzing the published mRNA expression profiles (GSE16515, GSE28735, and GSE15471) acquired from the Gene Expression Omnibus database, we discovered that the RHBDL2 mRNA was noticeably upregulated in PC tissues compared with that in normal/non-tumor tissues (Fig. S1A–C). The upregulation of RHBDL2 in PC tissues was also confirmed in the data obtained from The Cancer Genome Atlas (TCGA) database (Fig. S1D). Furthermore, analysis of PAAD datasets from TCGA database showed that high RHBDL2 expression in PC patients resulted in a shorter survival time (Figure S1E). Similarly, qRT-PCR and western blot assays indicated that RHBDL2 was upregulated at the mRNA and protein levels in all six human PC cell lines (CFPAC-1, MIA PaCa-2, SW1990, AsPC-1, PANC-1, and BxPC-3) compared with that in the primary normal human pancreatic duct epithelial (HPDE) cells (Fig. 1A, B). Concordantly, the expression level of RHBDL2 was examined by conducting qRT-PCR and western blot assays of PC tissues and adjacent non-tumor tissues, which showed that the RHBDL2 was upregulated in PC tissues (Fig. 1C, D). Furthermore, immunohistochemistry (IHC) assay also showed that the RHBDL2 expression was dramatically upregulated in PC tissues, especially in patients with distant metastasis (Fig. 1E, F). Moreover, the Kaplan-Meier plot indicated that PC patients with high RHBDL2 expression had a short survival time, whereas PC patients with low RHBDL2 expression had better survival (Fig. 1G). Taken together, these results suggest that RHBDL2 may have significant clinical value in PC.

### RHBDL2 promotes the proliferation, migration, and invasion of PC cells in vitro and in vivo

**Fig. 2:**
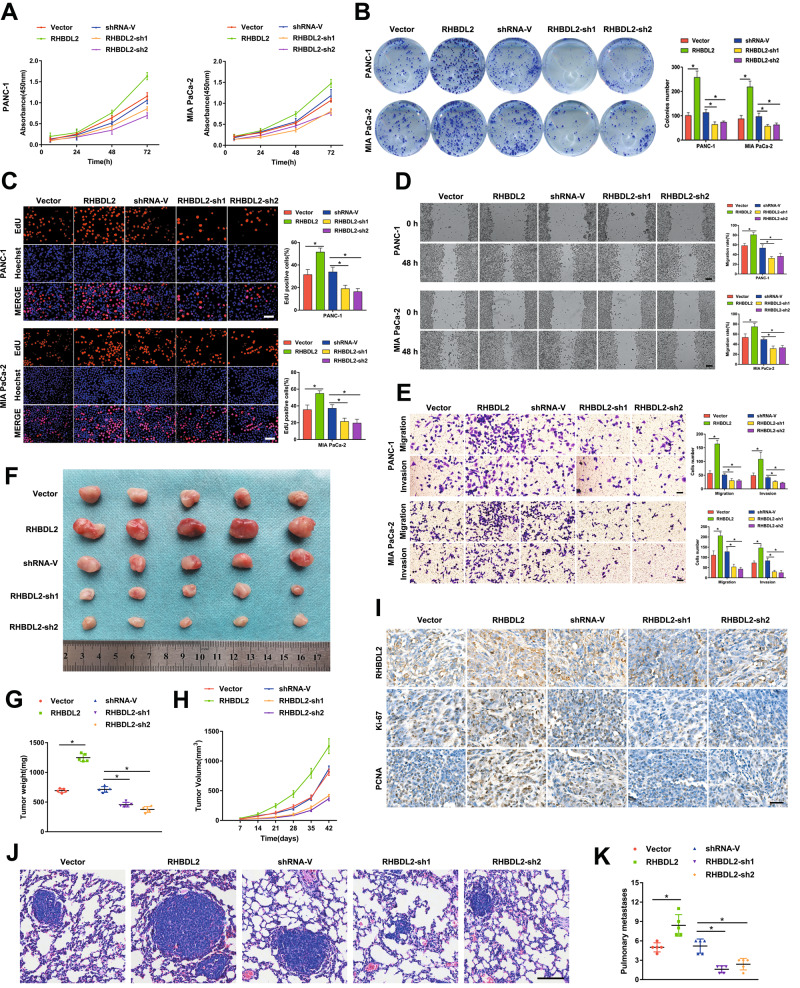
RHBDL2 promotes proliferation and mobility of PC cells in vitro and in vivo. CCK-8 (**A**), colony formation (**B**), and EdU (scale bar: 50 μm) (**C**) assays were used to evaluate the proliferation capacity of the RHBDL2-overexpressing, RHBDL2 downregulated, and corresponding negative control cells. The mobility of the indicated PC cells was verified by wound healing (scale bar: 100 μm) (**D**) and Transwell assays (scale bar: 50 μm) (**E**). Subcutaneous xenograft tumors (**F**), tumor weight (**G**), and tumor volume (**H**) in nude mice (*n* = 25). **I** IHC staining of RHBDL2 and the levels of Ki-67 and PCNA in the indicated tumors resected from the nude mice. Scale bar: 50 μm. Representative images of the tail vein-injected mouse models (scale bar: 100 μm) (**J**), and the lung foci number was evaluated (**K**), (*n* = 25). *P < 0.05.

To assess the biological function of RHBDL2 in PC cells, loss- and gain-of-function assays were performed. The results of CCK-8, colony formation, and EdU assays showed that RHBDL2-overexpressing PC cells had enhanced proliferation capacity, whereas the downregulation of RHBDL2 decreased the PC cell growth (Fig. 2A–C). As shown in the wound healing and Transwell assays, the upregulation of RHBDL2 confers the enhanced migratory and invasive abilities to PC cells. However, RHBDL2 silencing revealed a decrease in the mobility of these cells (Fig. 2D, E). To evaluate the impact of RHBDL2 on PC cells in vivo, a nude mouse xenograft tumor model was constructed. The upregulation of RHBDL2 expression facilitated tumor growth, while RHBDL2 silencing led to the inhibition of tumor growth (Fig. 2F). The RHBDL2 overexpression group showed higher tumor weight and volume, while the RHBDL2 knockdown groups had lower tumor weight and volume (Fig. 2G, H). Moreover, IHC staining showed high RHBDL2, Ki-67, and PCNA expression levels in tumor tissues with RHBDL2 upregulation. However, the RHBDL2-silenced groups showed lower RHBDL2, Ki-67, and PCNA levels (Fig. 2I). Furthermore, to verify whether RHBDL2 can accelerate metastasis in vivo, PC cells were injected in the tail vein of nude mice. The overexpression of RHBDL2 significantly increased the number of lung metastatic nodules, whereas RHBDL2 downregulation successfully weakened the degree of lung metastasis (Fig. 2J, K). Collectively, these findings suggest that RHBDL2 facilitates PC tumorigenesis and distant metastasis.

### RHBDL2 promotes the proliferation, migration, and invasion of PC cells by activating the Notch signaling pathway

**Fig. 3:**
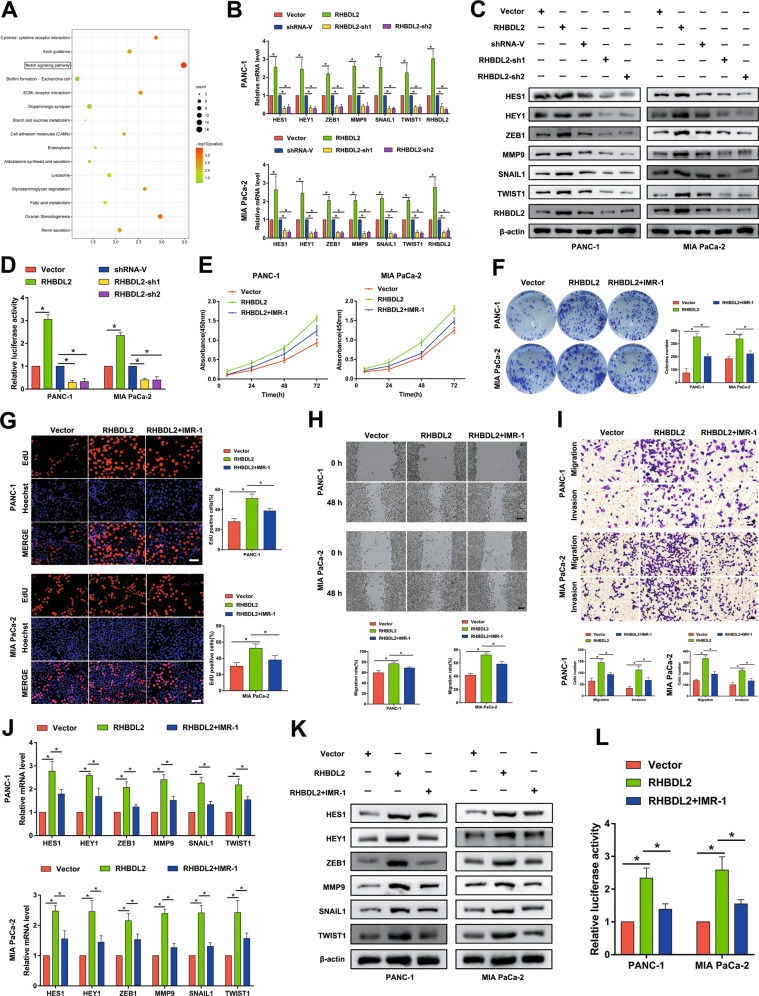
RHBDL2 promotes proliferation and mobility of PC cells by activating the Notch signaling pathway. **A** The result of RNA-seq analysis. **B**, **C** qRT-PCR and Western blot analyses of RHBDL2, HES1, HEY1, ZEB1, MMP9, SNAIL1, and TWIST1 in the indicated PC cells. **D** Dual Luciferase Reporter Assay. CCK-8 (**E**), colony formation (**F**), and EdU (scale bar: 50 μm) (**G**) assays of the PC cells incubated with/without IMR-1 (the inhibitor of Notch signaling pathway). Wound healing (scale bar: 100 μm) (**H**) and Transwell (scale bar: 50 μm) (**I**) assays of the indicated PC cells incubated with/without IMR-1. **J**, **K** qRT-PCR and Western blot analyses of HES1, HEY1, ZEB1, MMP9, SNAIL1, and TWIST1 in the indicated PC cells incubated with/without IMR-1. (**L**) Dual Luciferase Reporter Assay of the indicated PC cells incubated with/without IMR-1. *P < 0.05.

To evaluate which signaling pathway may be involved in the oncogenic effect of RHBDL2 in PC cells, a RNA-seq analysis was performed. As shown in Fig. 3A, RHBDL2 expression was significantly related to the activation of Notch signaling pathway. Consistently, the results of qRT-PCR and western blot assays showed that the expression level of RHBDL2 and the well-characterized downstream target genes hes family bHLH transcription factor 1 (HES1), hes related family bHLH transcription factor with YRPW motif 1 (HEY1), zinc finger E-box binding homeobox 1 (ZEB1), matrix metallopeptidase 9 (MMP9), snail family transcriptional repressor 1 (SNAIL1), and twist family bHLH transcription factor 1 (TWIST1) of the Notch signaling pathway were dramatically enhanced in the RHBDL2 overexpression group but decreased in the RHBDL2-silenced groups (Fig. 3B, C). In addition, RHBDL2 overexpression dramatically upregulated the luciferase reporter activity; however, knockdown of RHBDL2 led to the suppression of luciferase reporter activity (Fig. 3D). Next, the influence of the Notch signaling pathway on RHBDL2′s oncogenic role in PC cells was investigated by conducting rescue assays with the application of IMR-1, a small molecule inhibitor of the Notch signaling pathway. As shown in Fig. 3E–G, the results of CCK-8, colony formation, and EdU assays indicated that the Notch signaling pathway inhibitor IMR-1 blocked the RHBDL2-mediated enhanced proliferation capacity in PC cells. Similarly, wound healing and Transwell assays also showed that the migratory and invasive abilities of PC cells strengthened by RHBDL2 were partly reversed by the IMR-1 (Fig. 3H, I). Moreover, treatment of PC cells with IMR-1 abolished the effect of RHBDL2 on the expression level of Notch signaling pathway downstream target genes (HES1, HEY1, ZEB1, MMP9, SNAIL1, and TWIST1) (Fig. 3J, K), and on the luciferase reporter activity (Fig. 3L). The above results illustrate that RHBDL2 accelerates PC cell proliferation and motility by activating the Notch signaling pathway.

### RHBDL2 interacts with Notch1, mediates the cleavage of Notch1 and release of Notch1 intracellular domain

**Fig. 4:**
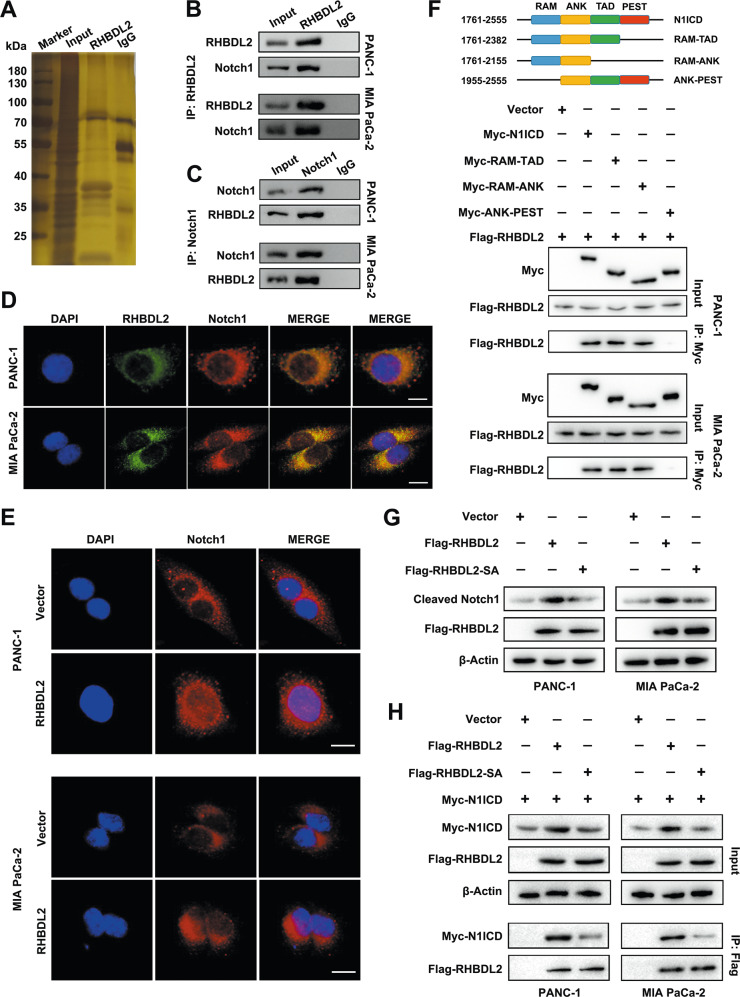
RHBDL2 interacts with Notch1 and mediates the cleavage of Notch 1 and release of Notch1 intracellular domain (N1ICD). **A** Silver staining assay was performed to detect the differential protein bands. **B**, **C** Immunoprecipitation (IP) assay of RHBDL2 and Notch1 in PC cells. Immunofluorescent (IF) staining assay presented the co-localization of RHBDL2 and Notch1 (**D**) and the distribution of Notch1 (**E**), respectively. Scale bar: 10 μm. **F** N1ICD’s domain organization and N1ICD truncations’ scheme, followed by IP assay. **G** The level of cleaved Notch1 was determined by Western blot assay. **H** IP assay detected the N1ICD level in the indicated PC cells. Flag-RHBDL2: RHBDL2 wild-type group; Flag-RHBDL2-SA: a serine to alanine (SA) catalytic mutant of RHBDL2.

Considering that RHBDL2 functions as an intramembrane serine protease, we hypothesized that RHBDL2 could bind to and subsequently cleave specific proteins in PC cells. To identify the potential binding partners of RHBDL2, whole-cell protein lysates were immunoprecipitated with RHBDL2 antibody. A silver staining assay was performed to determine the differential protein bands in the resultant immunoprecipitates. The lysate was analyzed using the MS approach. Results showed that Notch1 was the major binding partner of RHBDL2 (Fig. 4A). The interaction between RHBDL2 and Notch1 was determined in PC cells by IP assay (Fig. 4B, C). Furthermore, the colocalization of RHBDL2 and Notch1 was verified by immunofluorescence (IF) staining (Fig. 4D). IF staining also showed that the level of Notch1 increased in the nuclei of PC cells in the RHBDL2 overexpression group (Fig. 4E). The N1ICD domain was subsequently mapped to accurately detect the N1ICD-RHBDL2 interaction region. Results of the immunoprecipitation (IP) assay revealed that the N1ICD full-length, RAM-TAD, and RAM-ANK domains could pull down the RHBDL2, but the ANK-PEST domain could not (Fig. 4F). The upregulation of RHBDL2 led to a remarkable increase in the Notch1 and N1ICD levels, while RHBDL2 knockdown had the opposite effect. RHBDL2 enhanced the N1ICD levels in a dose-dependent manner (Figure S2 A–C). Western blot analysis indicated that the levels of cleaved Notch1 increased in RHBDL2 wild-type (Flag-RHBDL2) PC cells. When a serine to alanine (SA) catalytic mutant of RHBDL2 (Flag-RHBDL2-SA) was constructed, the elevated expression levels of cleaved Notch1 mediated by RHBDL2 was partially rescued (Fig. 4G). The IP assay results revealed that the N1ICD expression level was upregulated in the Flag-RHBDL2 group, while it was partly abolished in Flag-RHBDL2-SA PC cells in the input and IP groups (Fig. 4H). Taken together, our findings demonstrate that RHBDL2 interacts with Notch1 and mediates the cleavage of Notch1 and release of N1ICD through its intramembrane serine protease cleavage activity.

### RHBDL2 inhibits the degradation of N1ICD through the ubiquitin-proteasome pathway

**Fig. 5:**
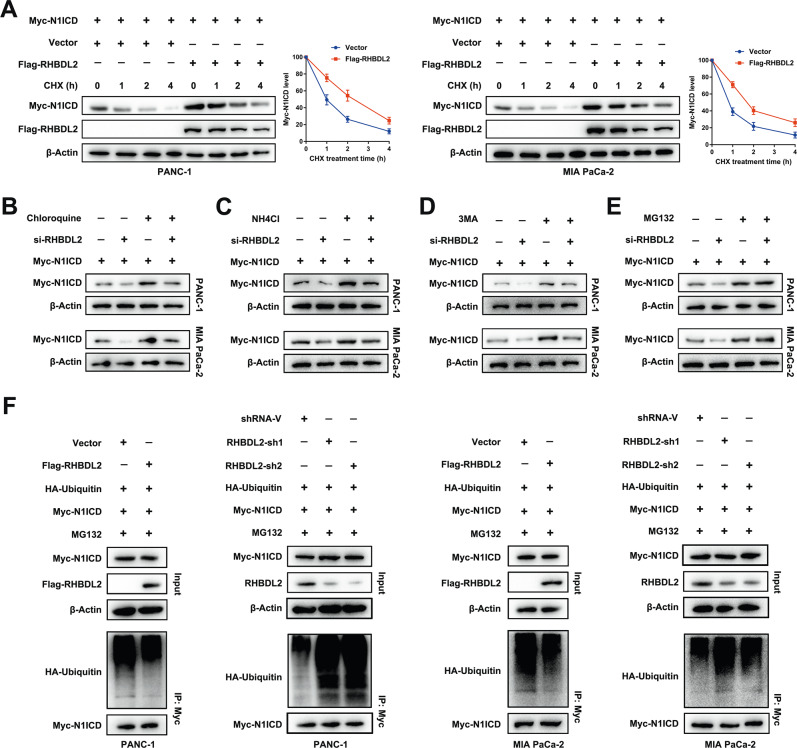
RHBDL2 inhibits the degradation of N1ICD via ubiquitin-proteasome pathway. **A** N1ICD and RHBDL2 levels were confirmed by Western blot analysis in the indicated PC cells treated with 40 μg/ml of cycloheximide (CHX). The indicated PC cells were treated with chloroquine (**B**), NH_4_Cl (**C**), 3-MA (**D**), or MG132 (**E**), respectively. Then, the level of N1ICD was detected by Western blot analysis. **F** The PC cells were treated with 15 μM of MG132 for 6 h prior to harvest. Cell lysate was immunoprecipitated with Myc-tag antibody and immunoblotted as indicated.

To determine whether RHBDL2 regulates N1ICD posttranslationally, the PC cells were treated with CHX to inhibit translation. N1ICD was rapidly degraded in the negative control group, while its protein half-life was prolonged in RHBDL2-overexpressing PC cells (Fig. 5A). The co-incubation of selective lysosomal inhibitors chloroquine and NH_4_Cl, or autophagy inhibitor 3-MA in PC cells could not recover the decreased N1ICD level mediated by RHBDL2 silencing (Fig. 5B–D). However, the co-incubation of PC cells with the proteasome inhibitor MG132 restored the decreased N1ICD level mediated by the downregulation of RHBDL2 (Fig. 5E). Moreover, results of the ubiquitination assay showed that RHBDL2 overexpression dramatically decreased the ubiquitination level of N1ICD. By contrast, the knockdown of RHBDL2 led to a significant increase in the N1ICD ubiquitination level (Fig. 5F). Collectively, these findings suggest that RHBDL2 inhibits the degradation of N1ICD through the ubiquitin-proteasome mechanism.

### RHBDL2 stabilizes the N1ICD through OTUD7B

**Fig. 6:**
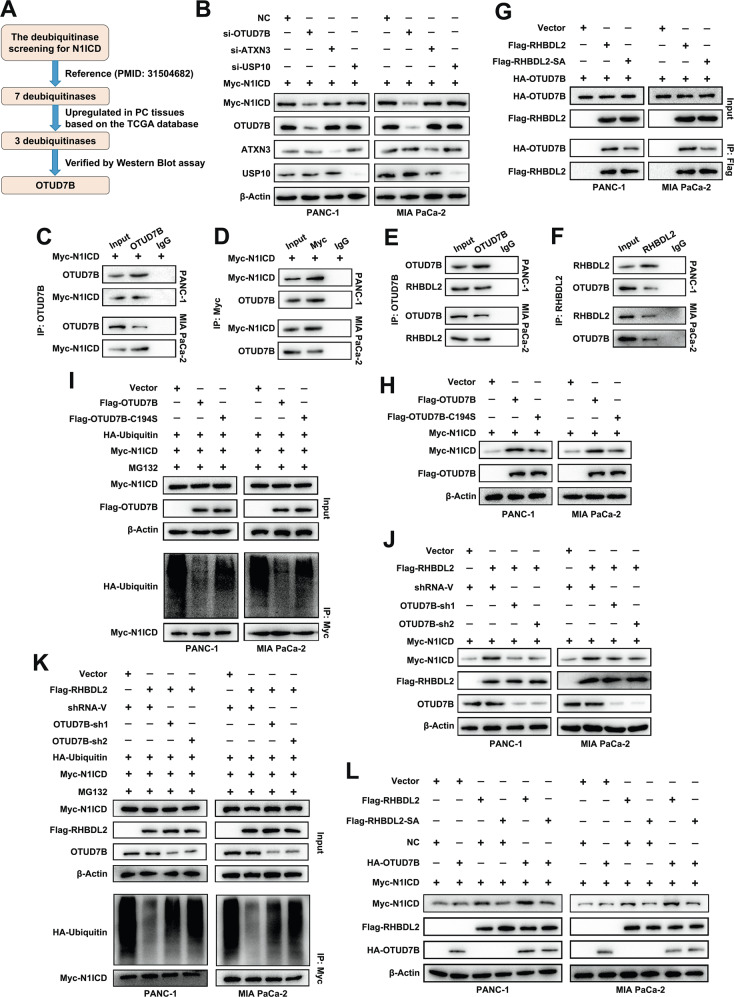
RHBDL2 stabilizes the N1ICD via the OTUD7B. **A** The deubiquitinase screening procedure for N1ICD. **B** Western blot analysis was performed to measure the expression levels of N1ICD, OTUD7B, ATXN3, and USP10 in the indicated PC cells. Immunoprecipitation assay of OTUD7B and N1ICD (**C–D**), OTUD7B and RHBDL2 (**E–F**). **G** The expression levels of HA-OTUD7B and Flag-RHBDL2 were confirmed by immunoprecipitation. **H** The levels of N1ICD and Flag-OTUD7B were determined by Western blot assay. **I** The PC cells were treated with 15 μM of MG132 for 6 h prior to harvest. The cell lysate was immunoprecipitated with Myc-tag antibody and immunoblotted as indicated. **J** The levels of N1ICD, Flag-RHBDL2, and OTUD7B in the indicated PC cells were detected by Western blot assay. **K** The PC cells were treated with 15 μM of MG132 for 6 h prior to harvest. The cell lysate was immunoprecipitated with Myc-tag antibody and immunoblotted as indicated. **L** The expression levels of N1ICD, RHBDL2, and OTUD7B in the indicated PC cells were determined by Western blot assay.

The RHBDL2 attenuates the ubiquitination level of N1ICD and stabilizes it via the ubiquitin-proteasome pathway, but the RHBDL2 has no DUB function. Thus, the present study identified the DUBs that could modulate N1ICD stability and interact with RHBDL2. According to a previous study [33], seven DUBs (ATXN3, BAP1, EIF3F, OTUD7B, UCHL5, USP7, and USP10) can regulate the stability of N1ICD. Furthermore, the expression levels of these seven DUBs were confirmed in the PAAD datasets from TCGA database (Fig. S3A–G). ATXN3, OTUD7B, and USP10 were highly expressed in the PC tissues (Fig. S3A, D, G). The N1ICD level was dramatically downregulated by OTUD7B silencing than by the knockdown of ATXN3 or USP10. Therefore, OTUD7B was selected for further analysis (Fig. 6A, B). IP assay results revealed that OTUD7B interacted with N1ICD (Fig. 6C, D) and RHBDL2 (Fig. 6E–F). The HA-OTUD7B expression levels remained the same in the Flag-RHBDL2-SA and Flag-RHBDL2 cells in the input group, but the level of immunoprecipitated HA-OTUD7B significantly decreased in the Flag-RHBDL2-SA cells compared with that in the Flag-RHBDL2 cells (Fig. 6G). The N1ICD level was profoundly upregulated in the OTUD7B wild-type (Flag-OTUD7B) group, but it was restored by the catalytically inactive mutant C194S of OTUD7B (Flag-OTUD7B-C194S) (Fig. 6H). Concordantly, the ubiquitination level of N1ICD was dramatically attenuated in the Flag-OTUD7B group, whereas the N1ICD ubiquitination level was restored in the Flag-OTUD7B-C194S group (Fig. 6I). Moreover, knockdown of OTUD7B in RHBDL2-overexpressing cells reversed the increase in N1ICD level and the reduction of N1ICD ubiquitination mediated by RHBDL2 (Fig. 6J, K). In addition, the upregulation of RHBDL2 in OTUD7B-overexpressing cells led to the profound increase in the N1ICD level, which was the highest among the other groups; however, the N1ICD level in the OTUD7B-overexpressing cells was restored by the Flag-RHBDL2-SA (Fig. 6L). Together, these findings suggest that RHBDL2 collaborates with OTUD7B to stabilize the N1ICD level through physical interactions.

### Association of the intramembrane serine protease cleavage activity of RHBDL2 and DUB function of OTUD7B with the oncogenic role of RHBDL2 in PC cells

**Fig. 7:**
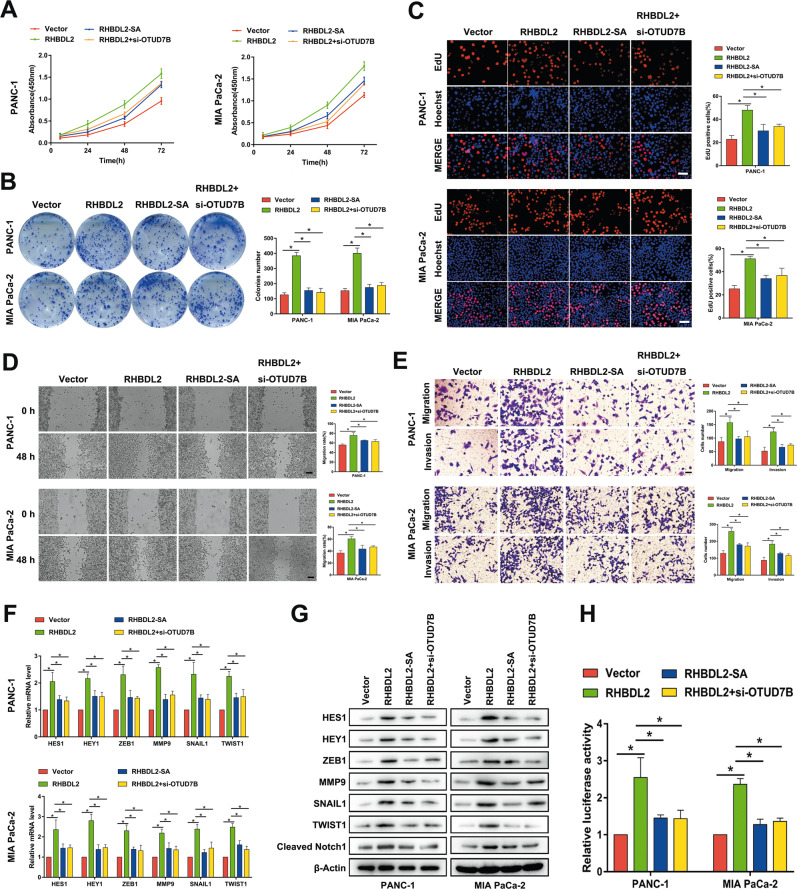
The oncogenic role of RHBDL2 in PC cells is dependent upon its intramembrane serine protease cleavage activity and the deubiquitinase function of OTUD7B. CCK-8 (**A**), colony formation (**B**), and EdU (scale bar: 50 μm) (**C**) assays were performed to determine the proliferation capacity in PC cells. The migration and invasion abilities of the indicated PC cells were evaluated by wound healing (scale bar: 100 μm) (**D**) and Transwell assays (scale bar: 50 μm) (**E**). **F**, **G** qRT-PCR and Western blot analyses of the Notch signaling pathway downstream target genes (HES1, HEY1, ZEB1, MMP9, SNAIL1, and TWIST1) and cleaved Notch1 in the indicated PC cells. (**H**) Dual Luciferase Reporter Assay of the indicated PC cells. *P < 0.05.

Next, we determined whether the intramembrane serine protease cleavage activity of RHBDL2 and the DUB function of OTUD7B were responsible for the RHBDL2-mediated Notch signaling pathway activation and facilitation of proliferation and mobility in PC cells. Mutation of the protease cleavage site of RHBDL2 or knockdown of OTUD7B in RHBDL2-overexpressing PC cells rescued their enhanced proliferation (Fig. 7A–C), migration, and invasion (Fig. 7D, E) capacity mediated by the RHBDL2. In addition, both the RHBDL2-SA and RHBDL2 plus OTUD7B-silencing groups significantly reversed the increased expression levels of the Notch signaling pathway downstream target genes (HES1, HEY1, ZEB1, MMP9, SNAIL1, and TWIST1) and cleaved Notch1 (Fig. 7F, G), as well as the enhanced luciferase reporter activity (Fig. 7H) mediated by the RHBDL2. These results suggest that the oncogenic role of RHBDL2 in PC cells is dependent on its intramembrane serine protease cleavage activity and the DUB function of OTUD7B.

## Discussion

The activity of the Notch signaling pathway is often altered in various human malignant tumors [34**–**36] including PC [37]. The Notch signaling pathway can accelerate the tumorigenesis of PC cells [17]. In the present study, the potential function and mechanism of RHBDL2 in the progression of PC were investigated, and results showed that it relied on the stabilization of N1ICD by RHBDL2 via the OTUD7B, which then activated the Notch signaling pathway.

First, to assess the significance of RHBDL2 in the development of PC, we investigated the expression levels of RHBDL2 and discovered that RHBDL2 was overexpressed in PC cell lines and tissue samples. The Kaplan-Meier survival curve revealed that RHBDL2 upregulation was closely correlated with the shorter survival time of patients with PC, indicating the significance of RHBDL2 in affecting the prognosis of PC patients. Subsequently, the overexpression of RHBDL2 was verified to promote PC cell proliferation and motility in vitro and in vivo using functional assays. Results of the RNA-seq analysis revealed that RHBDL2 expression was significantly correlated with the Notch signaling pathway. The expression levels of downstream target genes and transcriptional activity of the Notch signaling pathway increased upon the overexpression of RHBDL2. Furthermore, the role of RHBDL2 in the development of PC and the activation of the Notch signaling pathway mediated by RHBDL2 can be partially reversed by the Notch signaling pathway inhibitor IMR-1. These findings suggest that RHBDL2 may act as an oncogene by activating the Notch signaling pathway.

The Notch receptor binds to its ligand and subsequently undergoes a series of cleavage events induced by proteases, resulting in the production and release of the Notch intracellular domain (NICD). The translocation of NICD into the nucleus induces the activation of Notch signaling [15]. NUMB binds to N1ICD’s PEST domain and then stabilizes the N1ICD [33]. In our study, we demonstrated that RHBDL2 interacts with Notch1 and that N1ICD binds to RHBDL2 through its RAM domain. Furthermore, the overexpression of RHBDL2 enhanced the expression level of Notch1 in the nuclei of PC cells and upregulated the protein expression levels of cleaved Notch1 and N1ICD. The increase in cleaved Notch1 and N1ICD levels depends on the intramembrane serine protease cleavage activity of RHBDL2. In addition, RHBDL2 suppresses the degradation of N1ICD through the ubiquitin-proteasome pathway, thereby stabilizing the N1ICD.

As a DUB, OTUD7B removes the ubiquitin chains from proteins, thereby contributing to their stabilization [38]. OTUD7B can not only hydrolyze K11 ubiquitin chains but also decrease the K63-linked GβL ubiquitination level, thus facilitating the interaction between GβL and SIN1 and promoting the formation of mTORC2 to respond to a variety of growth signals [39]. Tang et al. found that OTUD7B expression is positively correlated with the ERα levels in breast cancer and can function as an independent factor for predicting the patient’s prognosis. Importantly, OTUD7B acts through a deubiquitination-dependent mechanism to stabilize the ERα [31]. OTUD7B is overexpressed in human metastatic or high-grade breast cancer; the dysregulation of OTUD7B correlates with worse survival and cancer metastasis. The OTUD7B is responsible for the deubiquitination of LSD1 at the K226/277 residues, and OTUD7B depletion increases the LSD1 K63‐linked ubiquitination and p62‐mediated proteolysis [40]. Zhang et al. reported that OTUD7B can bind to and deubiquitylate TRAF3, which results in the suppression of TRAF3 proteolysis and accumulation of NIK, thereby inactivating the non-canonical NF-κB signal and repressing lung cancer cell mobility induced by LCL161 [30]. Although accumulating studies have revealed that OTUD7B plays a pivotal role in tumor progression, the underlying molecular mechanism of OTUD7B in the development of PC remains unclear. In our current study, the N1ICD level is positively correlated with OTUD7B. OTUD7B interacts with N1ICD and RHBDL2 and stabilizes N1ICD through its DUB activity. The RHBDL2 collaborates with OTUD7B to block the degradation of N1ICD through their physical associations. Finally, rescue assays verified that the role of RHBDL2 in the development of PC is dependent on its intramembrane serine protease cleavage activity and the DUB function of OTUD7B.

In summary, our study illustrates that RHBDL2 acts as an oncogene in PC, stabilizes N1ICD through the OTUD7B, and activates the Notch signaling pathway, thereby accelerating PC cell proliferation and mobility. Thus, RHBDL2 may be a novel therapeutic target for patients with PC.

## Supplementary information

Supplementary figures and figure legends

Supplementary Table 1

Supplementary Table 2

The full and uncropped western blots

Reproducibility checklist

## Data Availability

The datasets used and/or analyzed during the current study are available from the corresponding author upon reasonable request.
