# Supplementary material for: RHBDL2 promotes the proliferation, migration, and invasion of pancreatic cancer by stabilizing the N1ICD via the OTUD7B and activating the Notch signaling pathway
Source: Cell Death Dis. 2022 Nov 9;13(11):945. doi: 10.1038/s41419-022-05379-3 (PMC9646733; doi:10.1038/s41419-022-05379-3)
Supplement: Supplementary file 1 — Supplementary figures and figure legends [file 41419_2022_5379_MOESM1_ESM.docx]

**
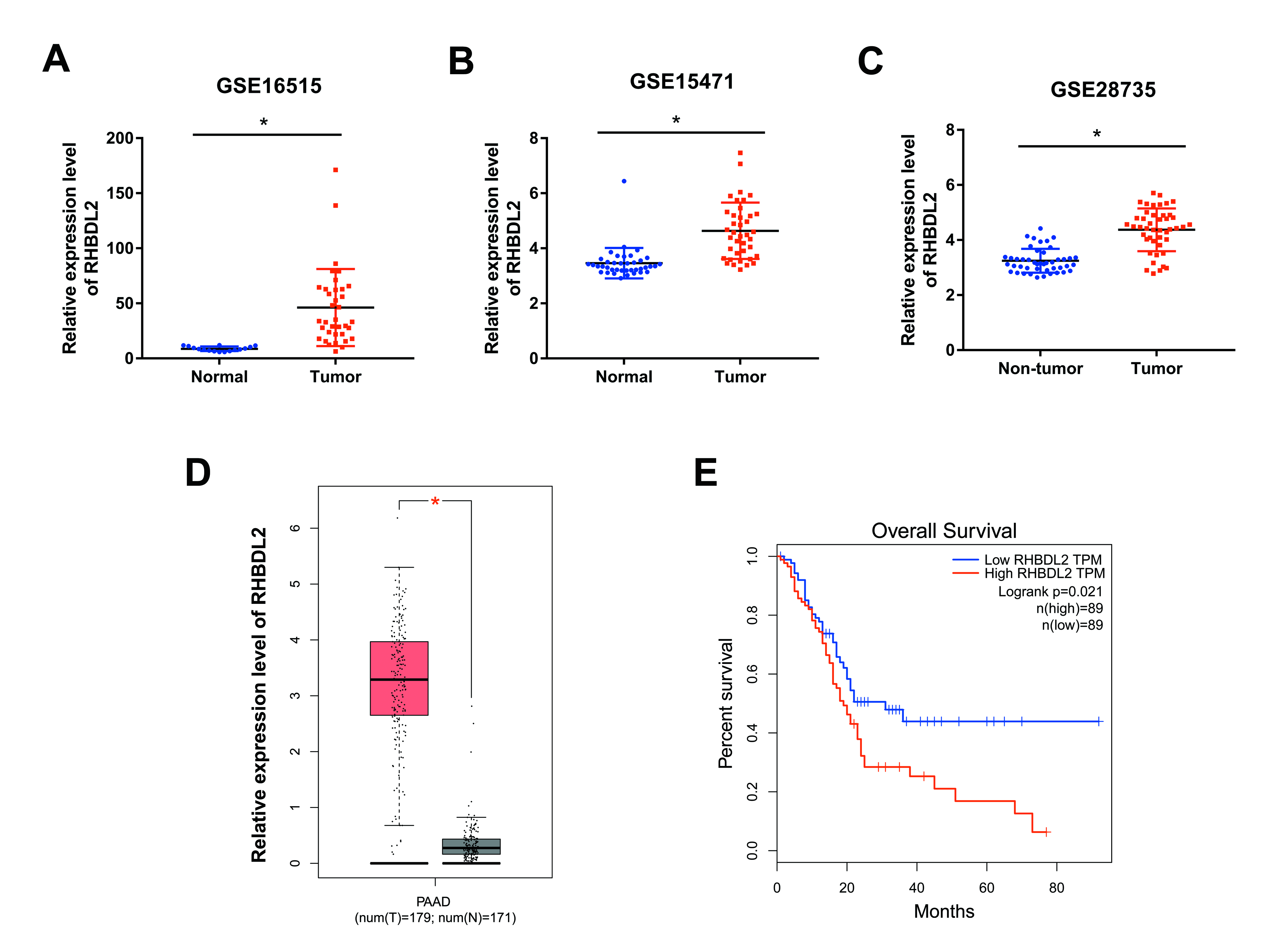
**

**Figure S1. RHBDL2 is overexpressed in PC tissues and positively related to poor prognosis of PC patients.** (**A–C**) Results of the expression profiling of mRNA showed that RHBDL2 is upregulated in PC tissues compared with that in normal/non-tumor tissues (GSE16515, GSE15471, and GSE28735). (**D**) The PAAD datasets obtained from TCGA database indicated that RHBDL2 is upregulated in PC tissues (T) compared with that in normal tissues (N). (**E**) Kaplan-Meier plot from the TCGA dataset of PC patients with low vs. high RHBDL2 expression levels. *P < 0.05


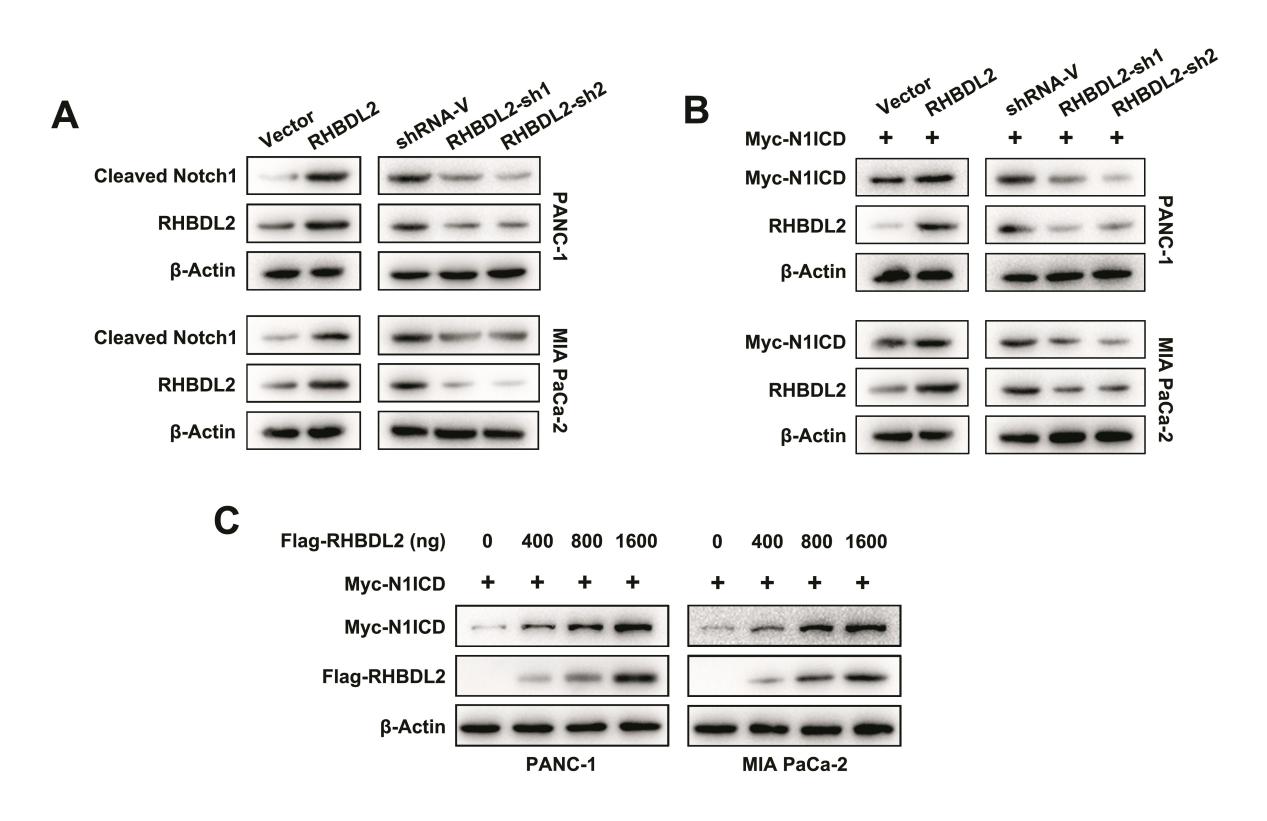


**Figure S2. RHBDL2 enhances the levels of cleaved Notch1 and N1ICD.** (**A–C**) The expression levels of cleaved Notch1, RHBDL2, and N1ICD in PC cells were confirmed by Western blot assay.


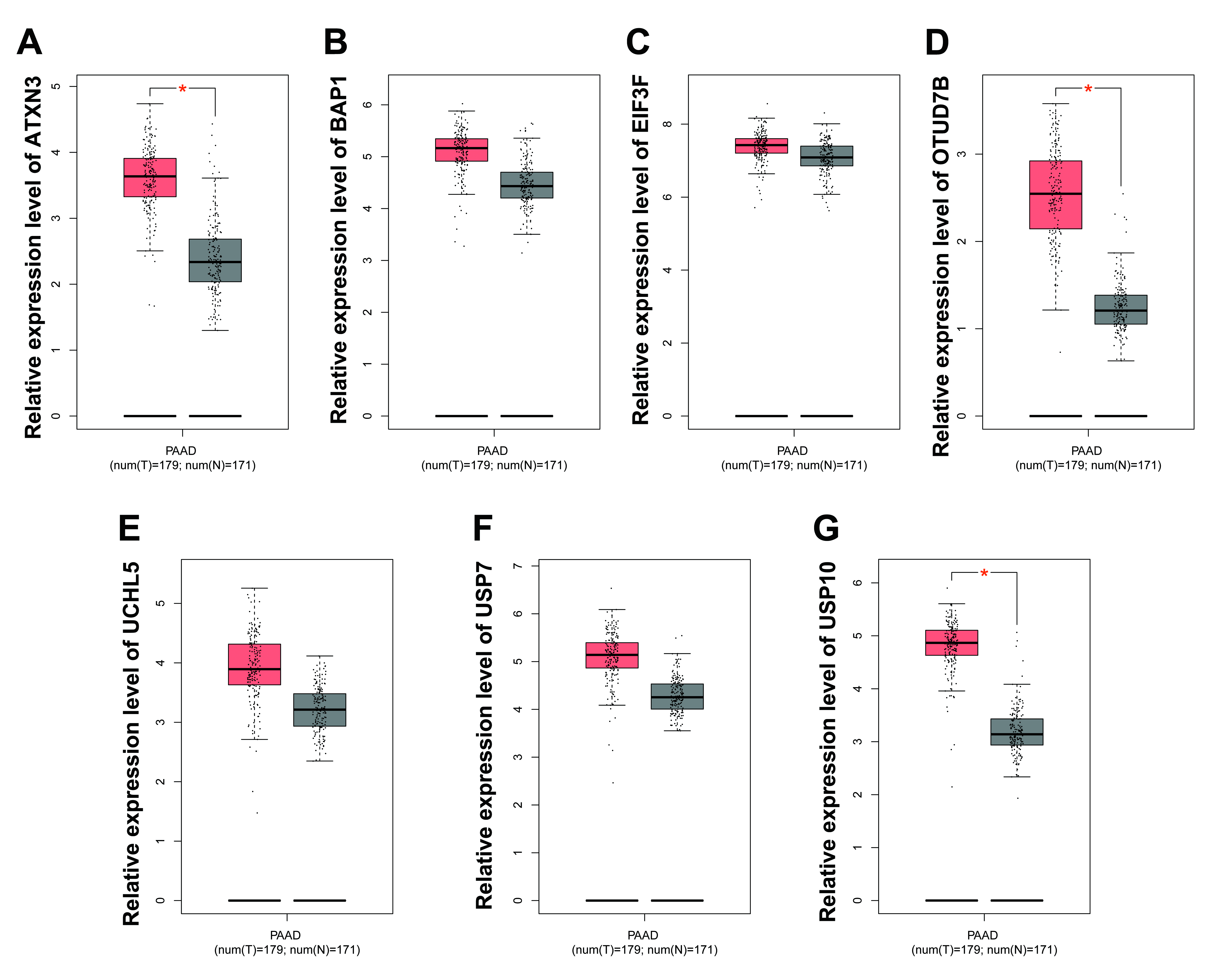


**Figure S3. Expression of seven deubiquitinases in PC.** (**A–G**) The relative expression levels of ATXN3 (**A**), BAP1 (**B**), EIF3F (**C**), OTUD7B (**D**), UCHL5 (**E**), USP7 (**F**), and USP10 (**G**) were verified in the PAAD datasets obtained from TCGA database. *P < 0.05

**
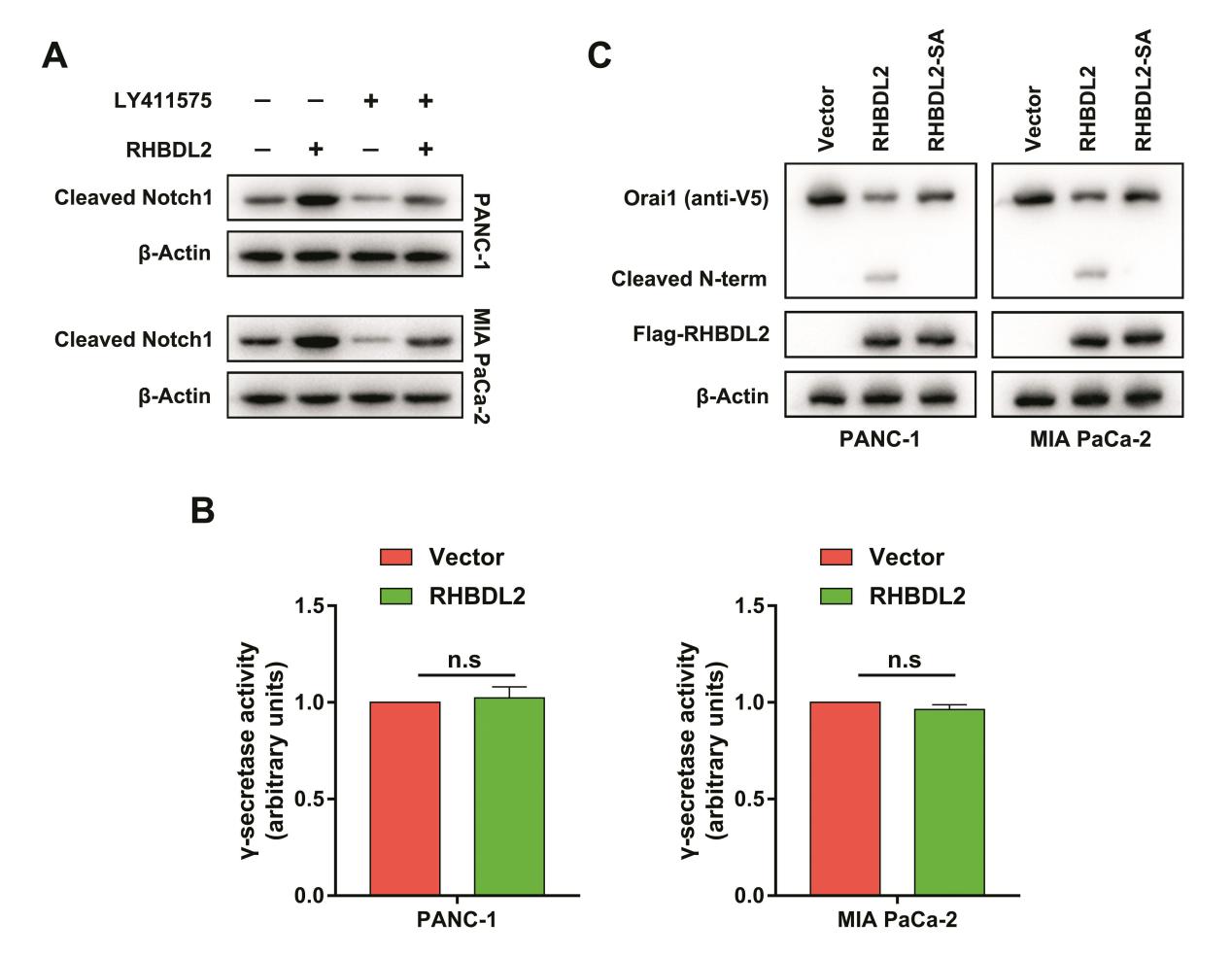
**

**Figure S4.**

(**A**) The cleaved Notch1 level was confirmed by Western blot analysis in the indicated PC cells treated with γ-secretase inhibitor LY411575.

(**B**) γ-Secretase Fluorogenic Substrate Assay was performed to detect the effect of RHBDL2 on γ-secretase activity in the indicated PC cells.

(**C**) The level of Orai1 and Flag-RHBDL2 was determined by Western blot assay in the indicated PC cells.
