# Supplementary material for: RHBDL2 promotes the proliferation, migration, and invasion of pancreatic cancer by stabilizing the N1ICD via the OTUD7B and activating the Notch signaling pathway
Source: Cell Death Dis. 2022 Nov 9;13(11):945. doi: 10.1038/s41419-022-05379-3 (PMC9646733; doi:10.1038/s41419-022-05379-3)
Supplement: Supplementary file 2 — Supplementary Table 1 [file 41419_2022_5379_MOESM2_ESM.docx]

**qRT-PCR Primers**

RHBDL2 (NCBI Gene ID :54933)

Forward Primer AAACAGTGGATCACGTTGGAC

Reverse Primer TTCCCCAAGATGTGCTGAACT

HES1 (NCBI Gene ID :3280)

Forward Primer TCAACACGACACCGGATAAAC

Reverse Primer GCCGCGAGCTATCTTTCTTCA

HEY1 (NCBI Gene ID :23462)

Forward Primer GTTCGGCTCTAGGTTCCATGT

Reverse Primer CGTCGGCGCTTCTCAATTATTC

ZEB1 (NCBI Gene ID :6935)

Forward Primer TTACACCTTTGCATACAGAACCC

Reverse Primer TTTACGATTACACCCAGACTGC

MMP9 (NCBI Gene ID :4318)

Forward Primer TGTACCGCTATGGTTACACTCG

Reverse Primer GGCAGGGACAGTTGCTTCT

SNAIL1 (NCBI Gene ID :6615)

Forward Primer TCGGAAGCCTAACTACAGCGA

Reverse Primer AGATGAGCATTGGCAGCGAG

TWIST1 (NCBI Gene ID :7291)

Forward Primer GTCCGCAGTCTTACGAGGAG

Reverse Primer GCTTGAGGGTCTGAATCTTGCT

β-actin (NCBI Gene ID :60)

Forward Primer CATGTACGTTGCTATCCAGGC

Reverse Primer CTCCTTAATGTCACGCACGAT
