# Supplementary material for: RHBDL2 promotes the proliferation, migration, and invasion of pancreatic cancer by stabilizing the N1ICD via the OTUD7B and activating the Notch signaling pathway
Source: Cell Death Dis. 2022 Nov 9;13(11):945. doi: 10.1038/s41419-022-05379-3 (PMC9646733; doi:10.1038/s41419-022-05379-3)
Supplement: Supplementary file 3 — Supplementary Table 2 [file 41419_2022_5379_MOESM3_ESM.docx]

| **No.** | **Name** | **Company** | **Catalog number** | **Dilution ratio** | **Molecular weight（kDa）** |
| --- | --- | --- | --- | --- | --- |
| 1 | RHBDL2 | proteintech | 12467-1-AP | WB : 1:1000  IHC: 1:200  IP : 2 ug  IF: 1:200 | 35 kDa |
| 2 | β-Actin | proteintech | 66009-1-Ig | WB : 1:30000 | 42 kDa |
| 3 | HES1 | CST | #11988 | WB : 1:1000 | 30 kDa |
| 4 | HEY1 | proteintech | 19929-1-AP | WB : 1:2000 | 33 kDa |
| 5 | ZEB1 | CST | #70512 | WB : 1:1000 | 200 kDa |
| 6 | MMP-9 | CST | #13667 | WB : 1:1000 | 92 kDa |
| 7 | SNAIL1 | proteintech | 13099-1-AP | WB : 1:1000 | 29 kDa |
| 8 | TWIST1 | proteintech | 25465-1-AP | WB : 1:1000 | 30 kDa |
| 9 | Notch1 | proteintech | 20687-1-AP | WB : 1:2000  IP : 2 ug  IF: 1:200 | 300 kDa |
| 10 | Cleaved Notch1 | CST | #4147 | WB : 1:1000 | 110 kDa |
| 11 | OTUD7B | proteintech | 16605-1-AP | WB : 1:1000  IP : 2 ug | 100 kDa |
| 12 | ATXN3 | proteintech | 13505-1-AP | WB : 1:1000 | 40 kDa |
| 13 | USP10 | proteintech | 19374-1-AP | WB : 1:1000 | 110 kDa |
| 14 | Ki-67 | proteintech | 27309-1-AP | IHC: 1:2000 |  |
| 15 | PCNA | proteintech | 10205-2-AP | IHC: 1:200 |  |
| 16 | IgG (Rabbit) | Beyotime | A7016 | IP : 2 ug |  |
| 17 | IgG (Mouse) | Beyotime | A7028 | IP : 2 ug |  |
| 18 | Myc-Tag | CST | #2276 | WB : 1:1000  IP : 1:1000 |  |
| 19 | DYKDDDDK Tag(Flag-tag) | CST | #14793 | WB : 1:1000  IP : 1:50 |  |
| 20 | HA-Tag | proteintech | 51064-2-AP | WB : 1:2000  IP : 2 ug |  |
| 21 | V5-Tag | CST | #13202 | WB : 1:1000 |  |
