# Supplementary figures and images for: RHBDL2 promotes the proliferation, migration, and invasion of pancreatic cancer by stabilizing the N1ICD via the OTUD7B and activating the Notch signaling pathway
Source: Cell Death Dis. 2022 Nov 9;13(11):945. doi: 10.1038/s41419-022-05379-3 (PMC9646733; doi:10.1038/s41419-022-05379-3)

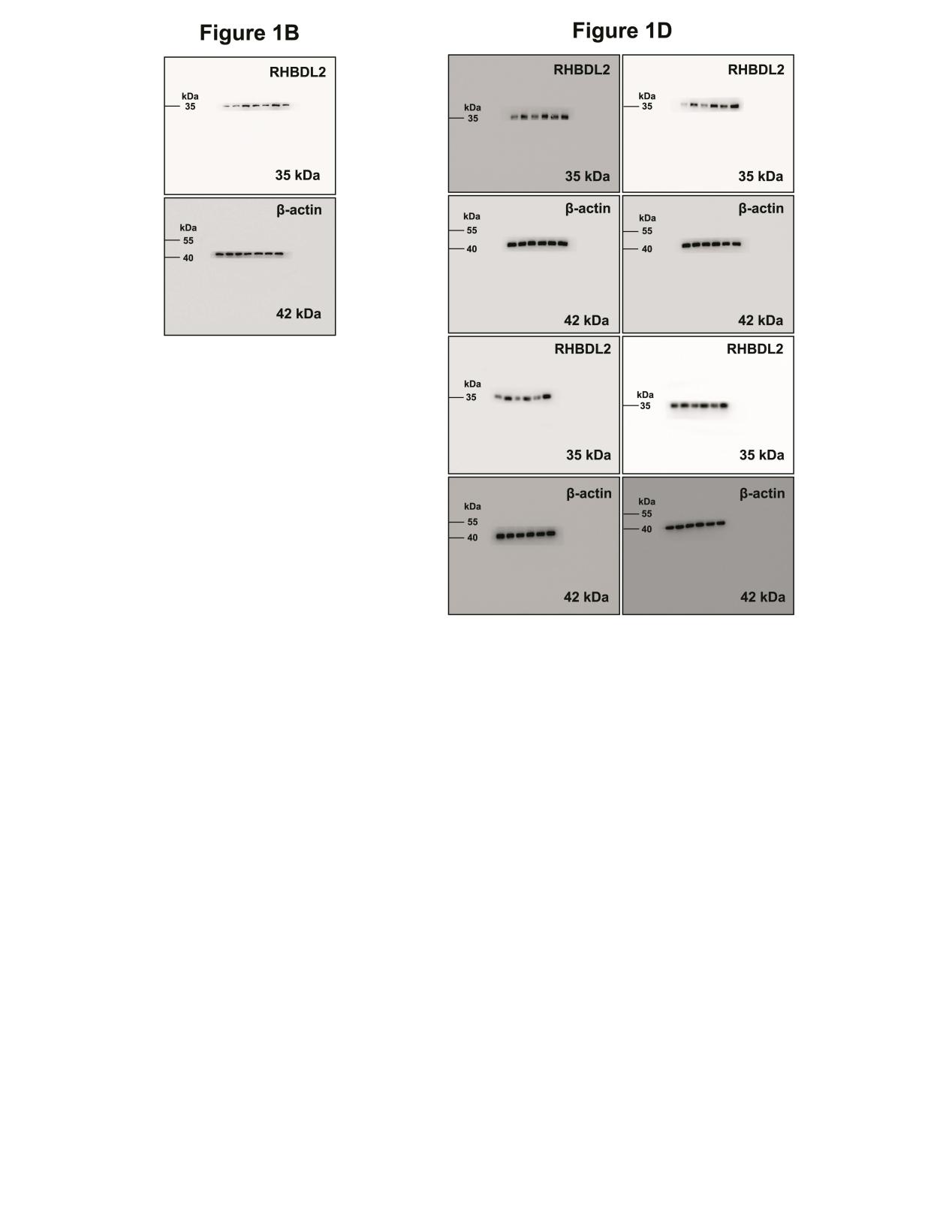


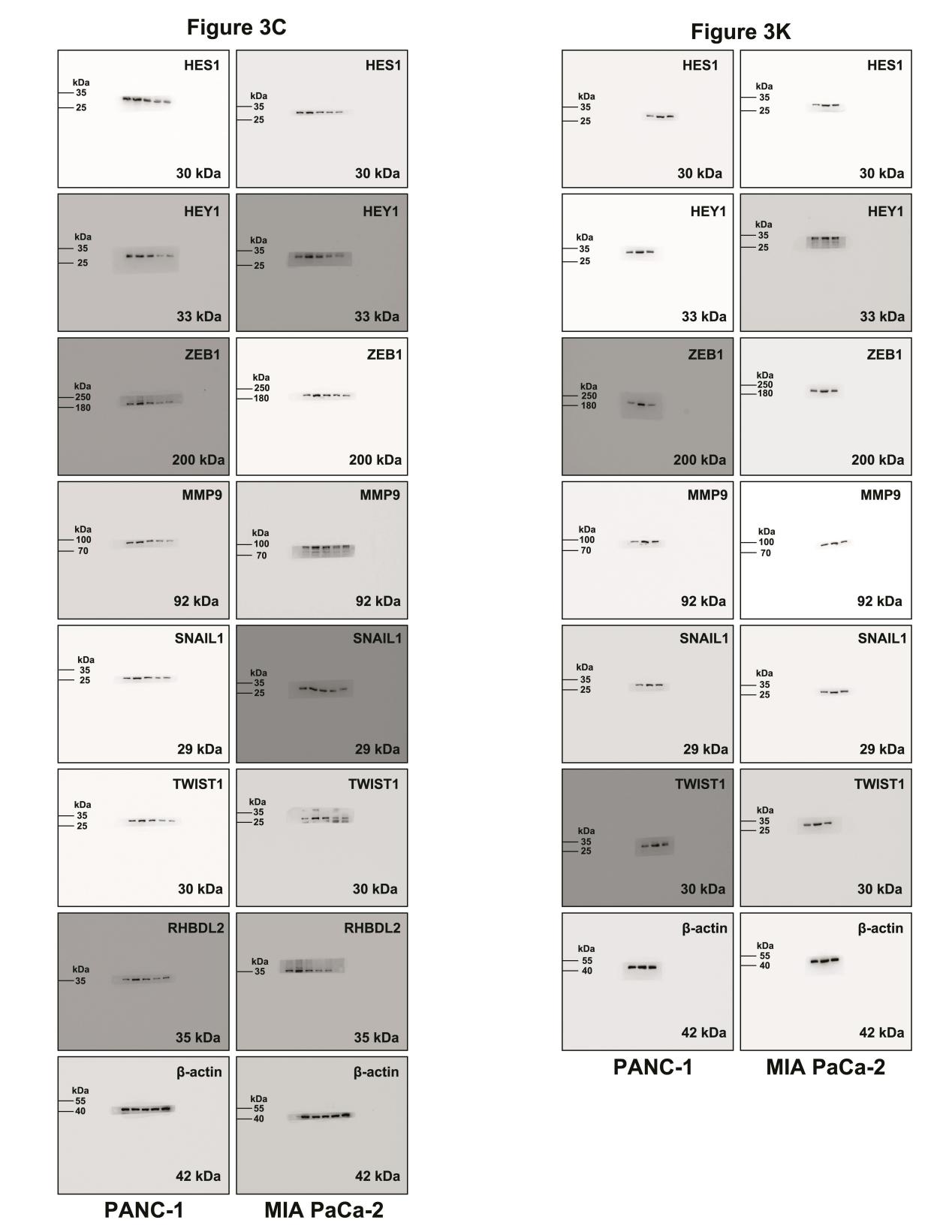


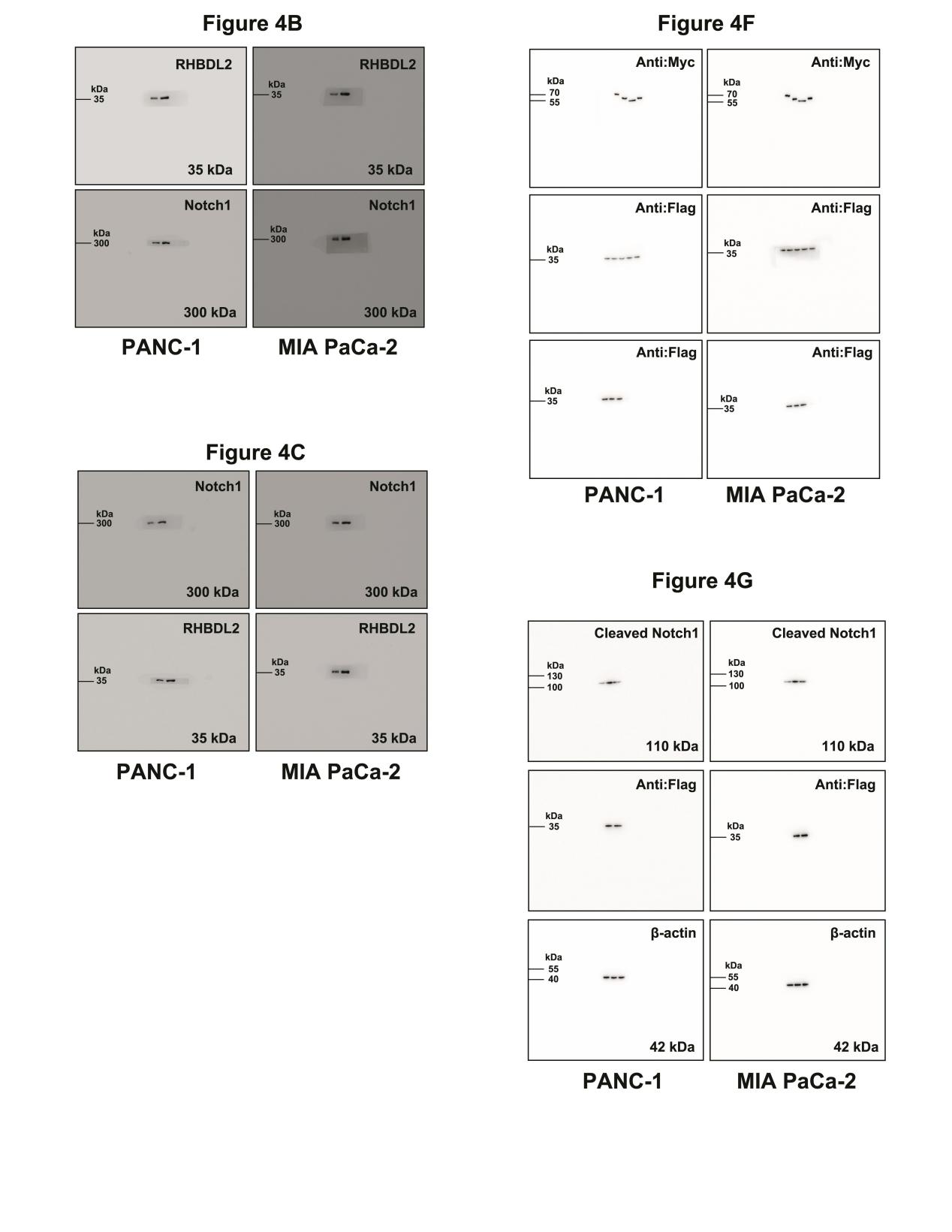


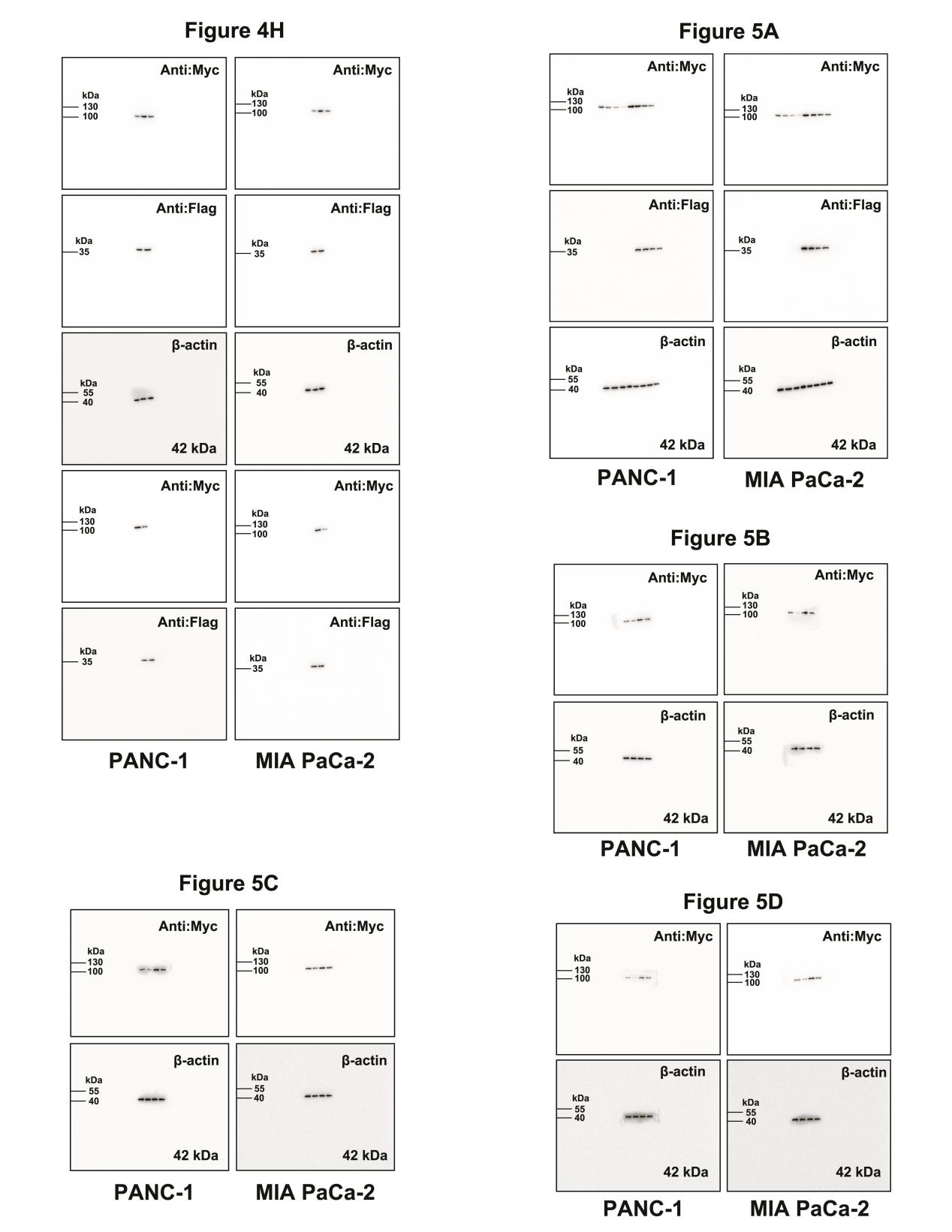


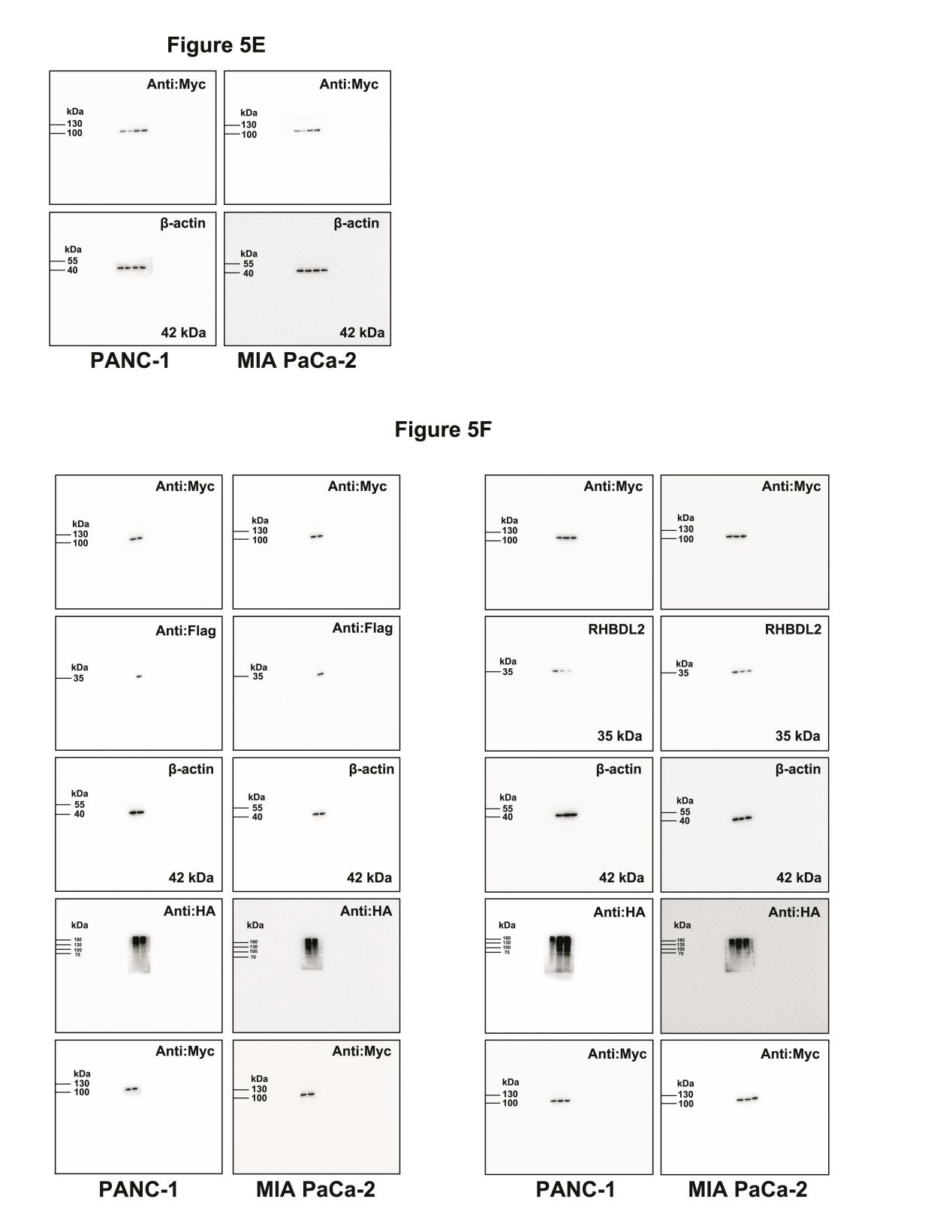


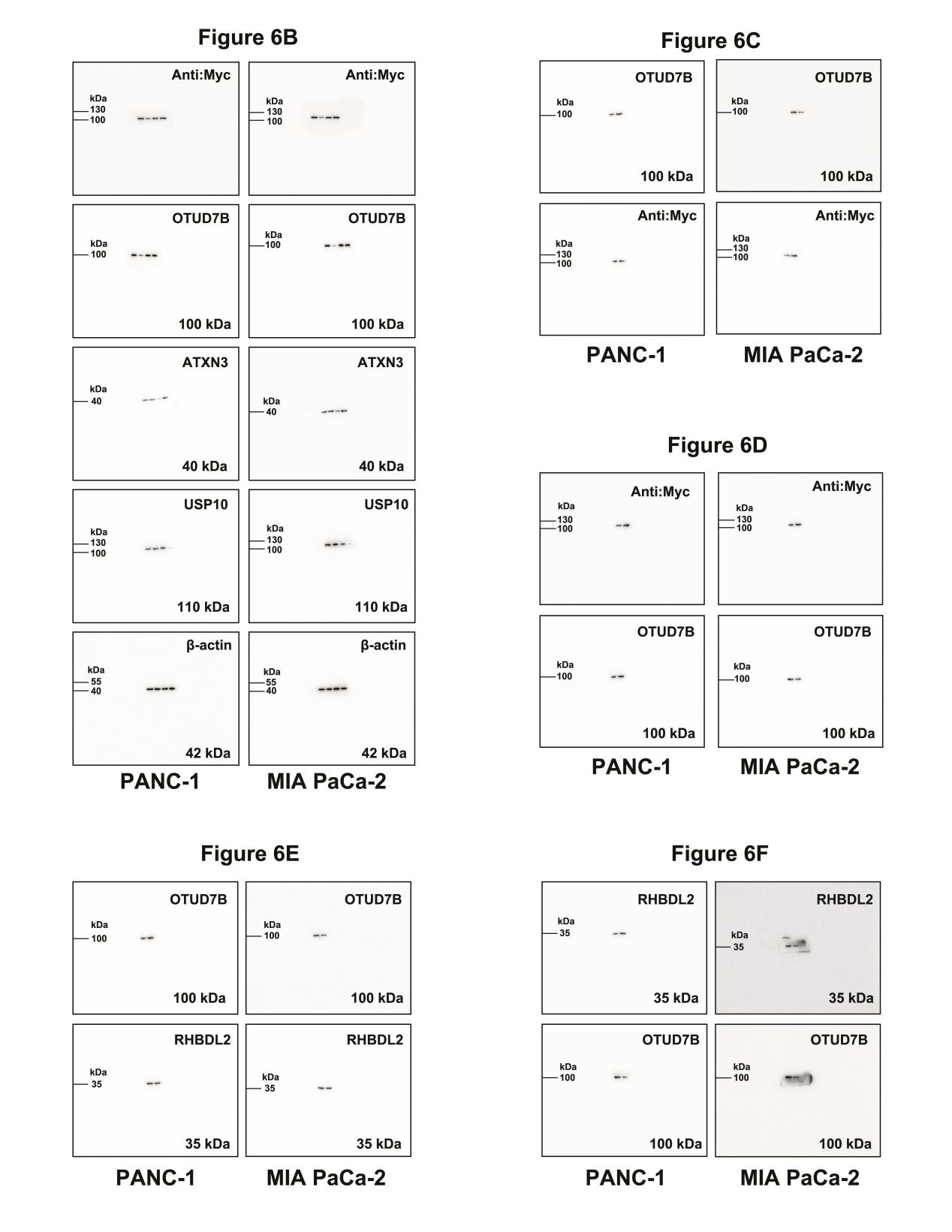


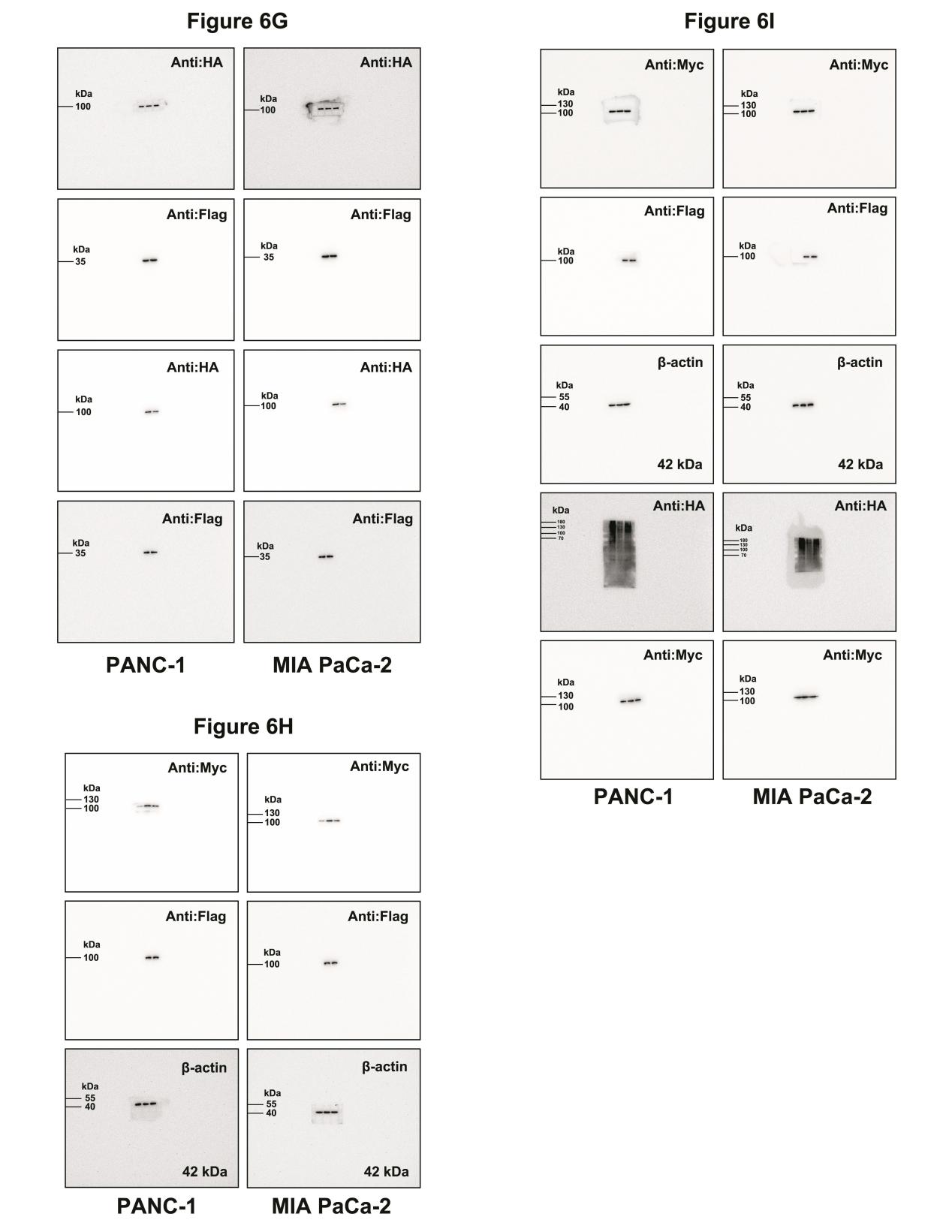


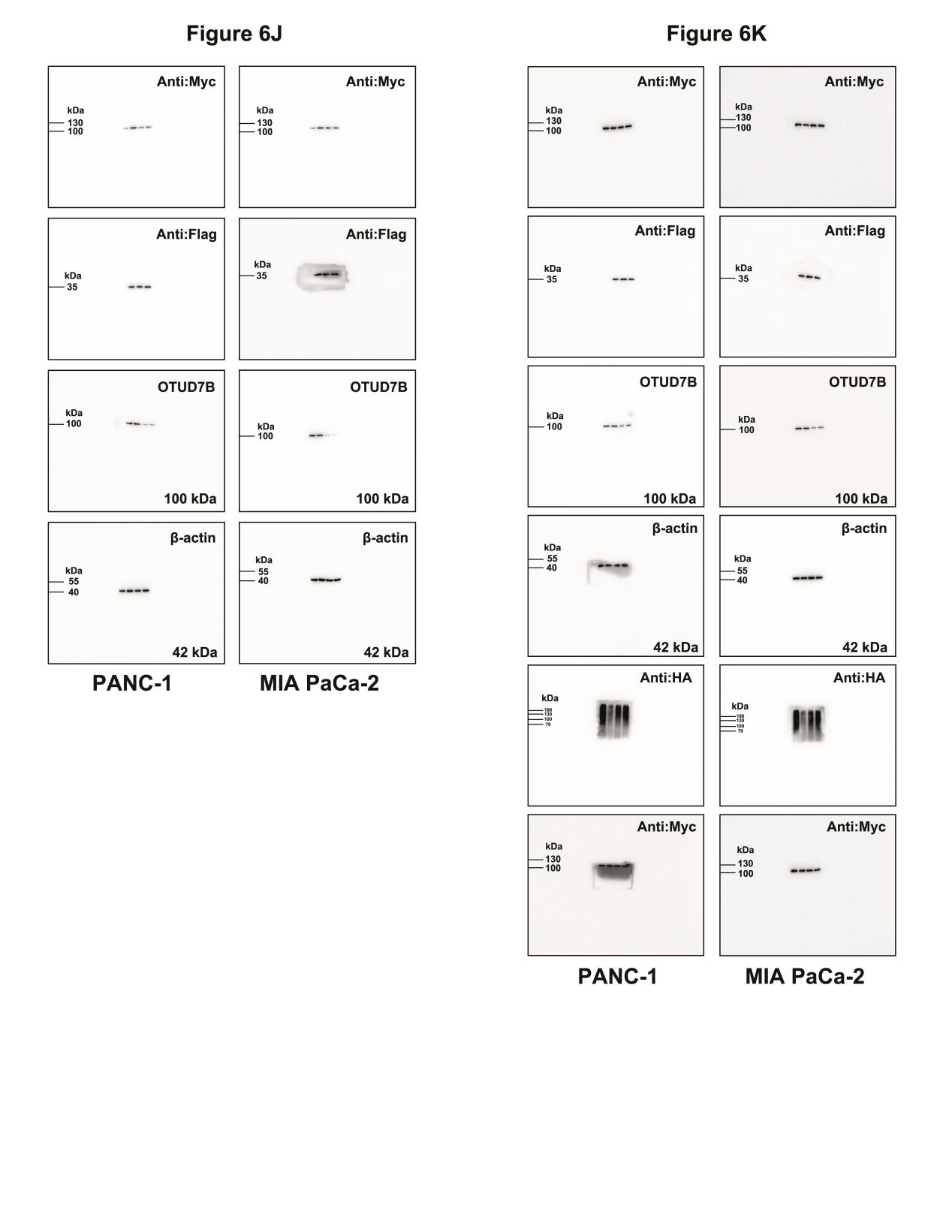


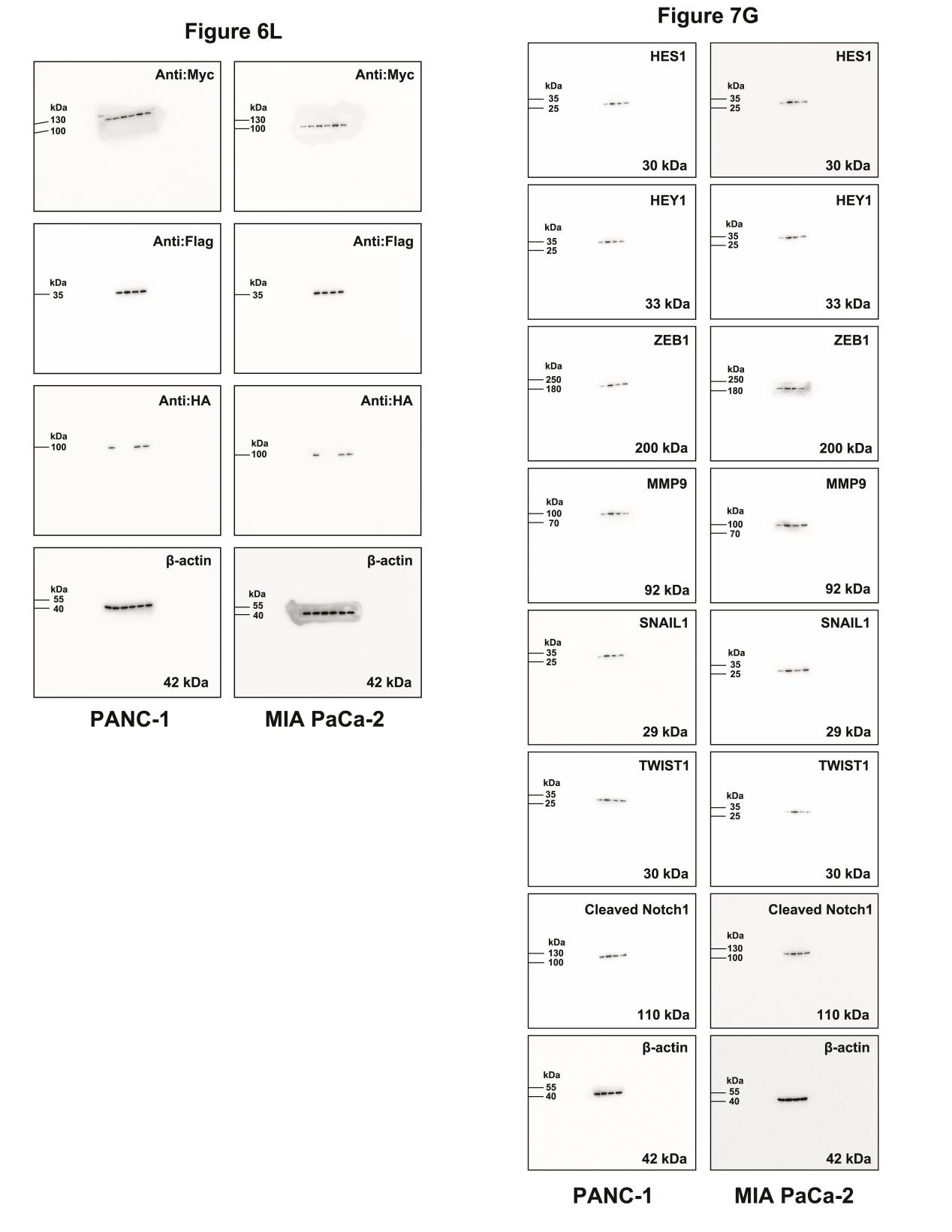


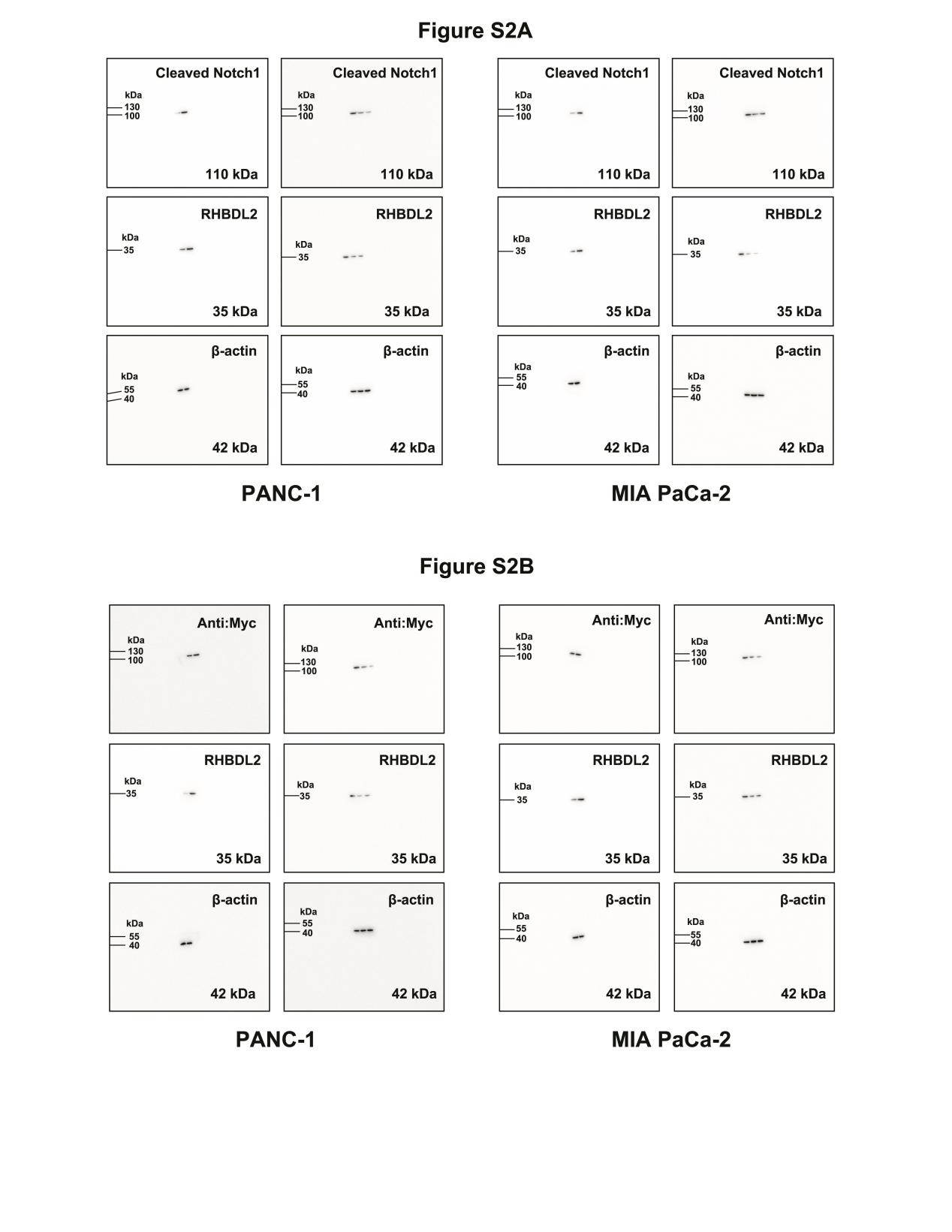


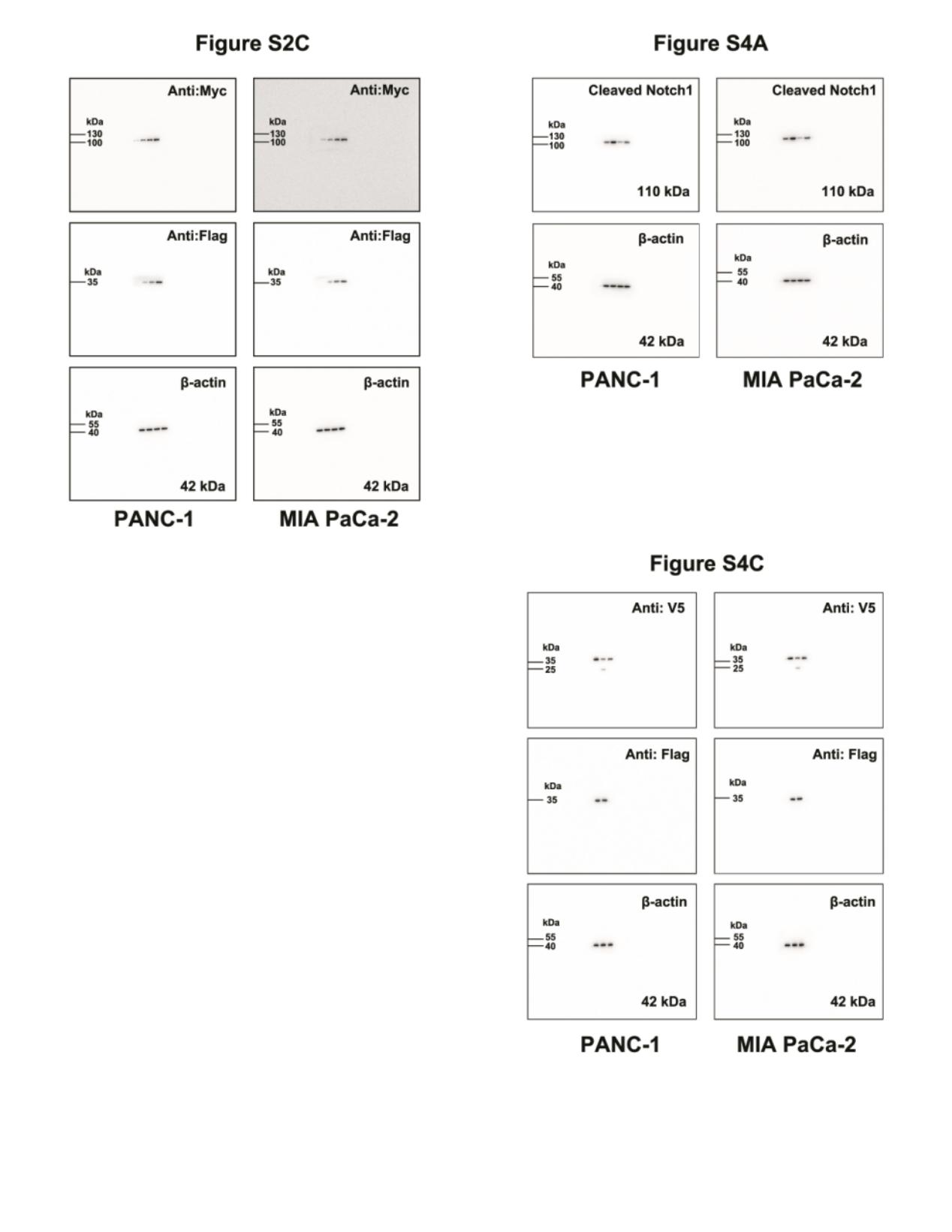

Supplement: Supplementary file 4 — The full and uncropped western blots [file 41419_2022_5379_MOESM4_ESM.docx]
